# Toward a Mobile Platform for Real-world Digital Measurement of Depression: User-Centered Design, Data Quality, and Behavioral and Clinical Modeling

**DOI:** 10.2196/27589

**Published:** 2021-08-10

**Authors:** Stefanie Nickels, Matthew D Edwards, Sarah F Poole, Dale Winter, Jessica Gronsbell, Bella Rozenkrants, David P Miller, Mathias Fleck, Alan McLean, Bret Peterson, Yuanwei Chen, Alan Hwang, David Rust-Smith, Arthur Brant, Andrew Campbell, Chen Chen, Collin Walter, Patricia A Arean, Honor Hsin, Lance J Myers, William J Marks Jr, Jessica L Mega, Danielle A Schlosser, Andrew J Conrad, Robert M Califf, Menachem Fromer

**Affiliations:** 1 Verily Life Sciences South San Francisco, CA United States; 2 Department of Computer Science Dartmouth College Hanover, NH United States; 3 Department of Psychiatry & Behavioral Sciences University of Washington Seattle, WA United States

**Keywords:** mental health, mobile sensing, mobile phone, mHealth, depression, location, GPS, app usage, voice diaries, adherence, engagement, mobility, sleep, physical activity, digital phenotyping, user-centered design

## Abstract

**Background:**

Although effective mental health treatments exist, the ability to match individuals to optimal treatments is poor, and timely assessment of response is difficult. One reason for these challenges is the lack of objective measurement of psychiatric symptoms. Sensors and active tasks recorded by smartphones provide a low-burden, low-cost, and scalable way to capture real-world data from patients that could augment clinical decision-making and move the field of mental health closer to measurement-based care.

**Objective:**

This study tests the feasibility of a fully remote study on individuals with self-reported depression using an Android-based smartphone app to collect subjective and objective measures associated with depression severity. The goals of this pilot study are to develop an engaging user interface for high task adherence through user-centered design; test the quality of collected data from passive sensors; start building clinically relevant behavioral measures (features) from passive sensors and active inputs; and preliminarily explore connections between these features and depression severity.

**Methods:**

A total of 600 participants were asked to download the study app to join this fully remote, observational 12-week study. The app passively collected 20 sensor data streams (eg, ambient audio level, location, and inertial measurement units), and participants were asked to complete daily survey tasks, weekly voice diaries, and the clinically validated Patient Health Questionnaire (PHQ-9) self-survey. Pairwise correlations between derived behavioral features (eg, weekly minutes spent at home) and PHQ-9 were computed. Using these behavioral features, we also constructed an elastic net penalized multivariate logistic regression model predicting depressed versus nondepressed PHQ-9 scores (ie, dichotomized PHQ-9).

**Results:**

A total of 415 individuals logged into the app. Over the course of the 12-week study, these participants completed 83.35% (4151/4980) of the PHQ-9s. Applying data sufficiency rules for minimally necessary daily and weekly data resulted in 3779 participant-weeks of data across 384 participants. Using a subset of 34 behavioral features, we found that 11 features showed a significant (*P*<.001 Benjamini-Hochberg adjusted) Spearman correlation with weekly PHQ-9, including voice diary–derived word sentiment and ambient audio levels. Restricting the data to those cases in which all 34 behavioral features were present, we had available 1013 participant-weeks from 186 participants. The logistic regression model predicting depression status resulted in a 10-fold cross-validated mean area under the curve of 0.656 (SD 0.079).

**Conclusions:**

This study finds a strong proof of concept for the use of a smartphone-based assessment of depression outcomes. Behavioral features derived from passive sensors and active tasks show promising correlations with a validated clinical measure of depression (PHQ-9). Future work is needed to increase scale that may permit the construction of more complex (eg, nonlinear) predictive models and better handle data missingness.

## Introduction

### Background

Mental health disorders are the leading cause of years lost to illness, disability, or premature death, as represented by disability-adjusted life years (DALYs) [1]. Perhaps, even more disquieting is the fact that we have made no progress in reducing these lost years over the past decades. From 1990 to 2016, years lived with disability (YLDs) for major depressive disorder, anxiety disorders, schizophrenia, dysthymia, and bipolar disorder have not changed, and the YLDs for opioid use disorders have even increased [2].

Although there are several reasons for this failure to improve outcomes, one area that has received increased attention is a relative lack of objective measurement in mental health [3]. Other fields have advanced by leveraging biomarkers and measurement tools to empower patients and clinicians to improve clinical decision-making. In the field of mental health, the absence of objective biomarkers leads to reliance on subjective information derived from clinical interviews and self-report surveys. Although the use of systematic self-report measures and subsequent algorithm-based treatment adjustments have shown significant promise [4,5], there is a substantial gap between the clinical trials that demonstrate this promise and what is deployed in routine practice [6]. A survey in the United Kingdom found that 80% of clinicians do not routinely use any outcome measures because they find them to be clinically irrelevant and too time consuming [7].

To address this shortcoming, the idea of taking advantage of the ubiquitous availability of smartphones has been proposed [8]. It stands to reason that novel data streams from digital sensors may provide a low-burden and scalable option to continuously quantify out-of-clinic real-world behaviors, which, in addition to traditional assessments in the clinic, could be used to bring psychiatry closer to realizing the consistently improved outcomes achieved by measurement-based care [9].

A number of studies have since explored the feasibility of linking smartphone or wearable data to clinical symptoms. In addition, passive sensing platforms have been built to collect smartphone data and examine the prediction of mental health clinical phenomena (Monsenso Monarca platform [10], Harvard Beiwe platform [11], Mindstrong [12], Dartmouth StudentLife platform [13], and Ginger.io [14], among others). A recent systematic meta-analysis review on the relationship between digital behavioral features and depressive mood symptoms concluded that this approach is promising [15]. However, most studies included in that review are not readily generalizable because of small sample sizes, where of the 26 studies of participants with mood disorders, all but one had fewer than 61 participants, and the median number of participants was 18. Furthermore, these studies were often designed as proof-of-concept investigations of single phone features (eg, actigraphy or GPS only). Even if meaningful correlations are found at this scale, the potential for identifying false-positive associations in situations where signals greatly outnumber clinical events cannot be ignored. Therefore, large-scale studies are necessary to adequately evaluate the associations and develop predictive models of clinical significance. A recent study examining the relationship coefficients between passive sensors and Patient Health Questionnaire (PHQ-2) for 271 individuals, for example, found that personalized models of behavioral features may be predictive of mood, with an area under the curve (AUC) of >0.5 for 81% of participants [16].

### Objectives

Therefore, we seek to create a scalable smartphone platform that can acquire robust, multisensor streams of passively collected signals from individuals with depression, in addition to longitudinal self-report survey measures. Here, we present the results of our first feasibility study with this platform app, where we aim to (1) deploy a user-friendly interface to achieve high study engagement among participants with depression while making enrollment and all study procedures fully remote and automated; (2) test the quality of collected passive and active data, including adherence to weekly PHQ-9 tasks; (3) demonstrate the ability to construct clinically relevant behavioral measures (*features*) from passive sensors and active inputs; and (4) preliminarily explore relationships between these features and symptoms of depression.

## Methods

### Overview

To achieve these objectives, we conducted a remote feasibility study with 600 participants. Of those participants, 80% (480/600) were treatment-seeking individuals with self-reported depression. The focus on treatment-seeking individuals was motivated by the assumption that recent treatment may lead to behavior and mood changes that can be captured during the course of the study. The remaining participants were nondepressed controls. Participants were also randomly assigned to one of the three compensation cohorts (Multimedia Appendix 1), allowing us to empirically test the effect of financial incentives on study adherence. We hypothesized that participants in the highest compensation group would show higher study adherence than those in the lower compensation groups.

**Figure 1:**
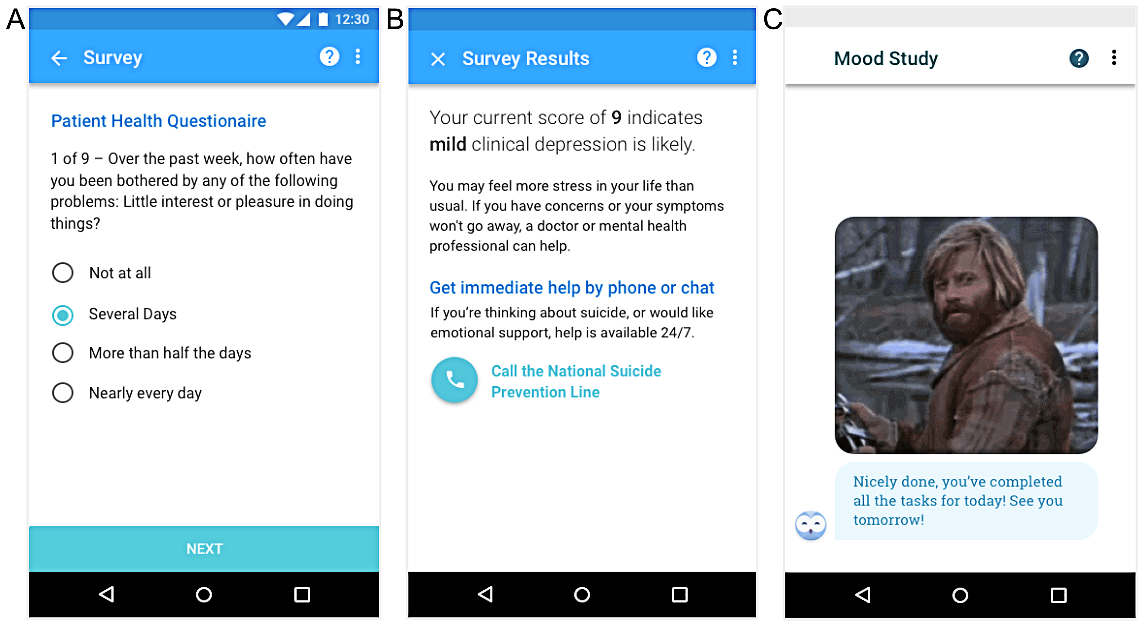
User interface of the study app. (A) Mobile rendering of the first question of the self-administered Patient Health Questionnaire-9 survey. (B) The score for the Patient Health Questionnaire-9 survey was tabulated and put into context for the study participant. (C) An owl avatar was used to deliver messages and occasional celebratory gif images.

The mobile app recorded data from smartphone-based sensors and provided a chatbot user interface (Figure 1) designed to engage participants and to collect self-report measures. The feasibility of launching a comprehensive sensor-based tool was evaluated via an examination of data quality and missingness of sensor and survey data as well as whether we would succeed in deriving behavioral features from the recorded raw sensor data. For an exploratory analysis, we hypothesized that location-based and social features would be related to mood symptoms, given previous literature citing such relationships. Another exploratory aim of the study was to build a multivariate model to discriminate between participants with and without depression using the derived behavioral features.

### Informed Consent

This study was approved by the Western Institutional Review Board Inc. In an effort to maximize transparency and mitigate privacy concerns, the informed consent form (ICF) included detailed descriptions of all sensors and the type of information that could be derived from them (Multimedia Appendix 2 provides a full copy of the ICF). It also included information about the relationship between Verily Life Sciences and Google and stated that data would not be shared with Google for advertising purposes. The app further featured a persistent notification (ie, it was visible at all times) prominently reminding the user that the app was continuously collecting data.

### Participants

Participants were recruited through social media and search engine advertisements (Facebook and Google AdWords). Our goal was to enroll 600 participants, 480 of which would be treatment-seeking individuals with depression and 120 nondepressed controls. In the cohort with 480 participants, participants had to have a PHQ-9 score of ≥10 (classified as *depressed* at enrollment). For the control group, participants were only included if their PHQ-9 score was <10 (classified as *not depressed* at enrollment). Participants were excluded when a plan to inflict self-harm was reported. The full list of inclusion and exclusion criteria can be found in the ICF in Multimedia Appendix 2.

### Study Design

#### Surveys

During the course of the 12-week study, participants provided active survey data in the form of daily questions about their sleep, functioning, and activity level. Participants were further asked to complete the PHQ-9, a quality of life questionnaire, and a voice diary on a weekly basis. Once a month, participants completed the Study Life Satisfaction scale [17] and the National Institute of Health Toolbox Loneliness scale [18], and participants completed a user feedback questionnaire after 6 weeks and 12 weeks in the study.

One subaim of this study was to determine an appropriate level of compensation that would sufficiently motivate participants to adhere to this intense survey protocol. To that end, tasks were compensated with different amounts depending on a random assignment to three compensation cohorts of US $135, US $265, or US $530 as the maximum study compensation if all tasks were completed. Participants were mailed a physical gift card, which was reloaded every 4 weeks with the accrued sum of their cohort-specific compensation. Further details of this subexperiment and the results can be found in Multimedia Appendix 1.

#### Sensors

We developed a mobile phone app for the Android operating system containing a broad range of *sensors* recording information from and about the following: accelerometer, ambient audio, Android device information, barometric air pressure, battery charge, Bluetooth, gyroscope, light level, magnetometer, network, phone calls, physical activity level, ping (a data point sampled every minute when the app was running), proximity (measures whether any object is within a given distance), screen state, step count, text messages, volume, and Wi-Fi networks. The ICF in Multimedia Appendix 2 provides more information and privacy statements about these sensors. Note that these sensors ranged from actual hardware sensors (eg, ambient audio) to measurements that rely on algorithmic processing of raw data (eg, inferring physical activity from raw accelerometer and gyroscope data).

### Data Filtering and Aggregation

Quality control for the data consisted of a stepwise process that considered a day’s worth of data for each participant and then considered a week’s worth of data. In particular, participant-days were included in the analysis if the following two conditions were met: (1) there were at least 720 minutes (ie, half a day’s worth) of available data for at least one of the sensors programmed to be sampled at regular 1-minute intervals (ie, ambient audio, ambient light, physical activity, location, pressure, ping, and proximity) and (2) the app had been active at least once during 18 different hours of the day. Subsequently, participant-weeks were included if there were 3 or more such days available to aggregate. In that case, the data for both regularly sampled features (eg, location) and user-driven features (eg, text messages and phone calls) were aggregated to a weekly mean. Note that using this rule, it is possible that a *specific feature* has fewer than 3 days or even no data at all available to aggregate (refer to the *Results* section).

### Feature Engineering

The next step in our data processing pipeline consisted of deriving clinically interpretable weekly summaries of the available underlying data, designated as weekly *behavioral features*. For some features (eg, ambient audio, ambient light, app use, and battery charge), aggregating average daily levels to average weekly levels was the only step of feature engineering. For other types of features, additional preprocessing was required. For example, to extract physical activity minutes, an algorithm determined the type of activity from a predefined set (eg, running, walking, and biking) from the accelerometer and gyroscope sensors. For voice diaries, algorithms were developed to extract features of interest from the audio file (eg, average pause duration and duration of diary) as well as from transcribed text (eg, spoken words per minute and sentiment score). Text messages (outgoing messages only) were also analyzed for their sentiment score, word and emoji count, and daily message count. Location data, originally collected as longitude and latitude every minute, were passed through a density-based spatial clustering of applications with noise (DBSCAN) clustering algorithm to arrive at location clusters. These clusters were further augmented using time-bounded heuristics to define "home" (the single place where participants spent the most time between 11 PM and 4 AM; this was required to be the same place over the 12 weeks of the study), "work" (any place where participants spent at least 15% of their time between 10 AM and 3 PM and which is not the "home" cluster; this could be multiple places over the course of the study), and "commute" (time spent traveling between home and work). The weighted average of latitudes and longitudes for each cluster was then used to retrieve semantic information from Google Maps about the type of location (eg, hospital or doctor’s office and place to exercise). Using this method, it is possible that a given central point is tagged with multiple types of location.

### Feature Selection

Feature engineering, as described earlier, yielded approximately 600 possible features. With a data set of only 384 participants, we decided a priori to use only 34 of those features, based on previous literature as well as clinical usefulness and interpretability.

### Statistical Analysis

#### Univariate Correlation Analysis

Pairwise correlations between the behavioral features and PHQ-9 were calculated on all weeks that had both the feature of interest and a weekly PHQ-9 survey available after applying the daily and weekly filtering rules. A total of 34 univariate tests were conducted, and to account for multiplicity, we used the Benjamini-Hochberg (nonnegative) procedure to adjust these 34 *P* values. The Python function *statsmodels.stats.multitest.multipletests* was used.

#### Multivariate Logistic Regression Model

Preprocessing for the multivariate model included standardizing all features using the *sklearn.preprocessing.StandardScaler* function. To predict the binarized PHQ-9 score (<10 or ≥10) from the 34 behavioral features, we fit an elastic net penalized logistic regression model with the function *sklearn.linear_model.LogisticRegression*. The inverse regularization parameter C was set to the default value of 1.0, and the L1 ratio (the elastic net mixing parameter) was set to 0.5. This model was passed through a 10-fold cross-validation procedure in which each participant’s data for all participant-weeks were placed in either the training or test set in each fold to prevent any label leakage.

## Results

### User-Centered Design

Previous studies examining the feasibility of digital measurement tools have been hampered by relatively high dropout rates. For example, in a randomized controlled trial on digital sensing and cognitive behavioral therapy on 1098 depressed participants, adherence dropped to 41% by the 12-week mark [19]. A similar study reported that only 10.5% of the initial 126 participants were still active at 8 weeks [20]. Therefore, another aim of this study was to design the user experience such that interaction with the app would be self-explanatory, engaging, and enjoyable.

We conducted a number of user experience studies on depressed participants and clinical professionals to design a user interface that would be appropriate in tone for participants with depression yet maximally engaging (for a detailed description of these studies, refer to Multimedia Appendix 3).

On the basis of this research, it was ultimately decided that our app would feature a chatbot interface that guides the user through onboarding and their daily survey tasks with a series of static, predefined messages and provides unexpected moments of delight using celebratory gif images (Figure 1). The chatbot was kept deliberately lighthearted, where appropriate, and conveyed gratitude for contributing to the overall mission. Task completion was immediately followed by a celebratory moment (text encouragement plus occasional gif image) and reward (information about how much compensation was earned for completing those tasks).

### Enrollment Funnel and Participant Characteristics

**Figure 2:**
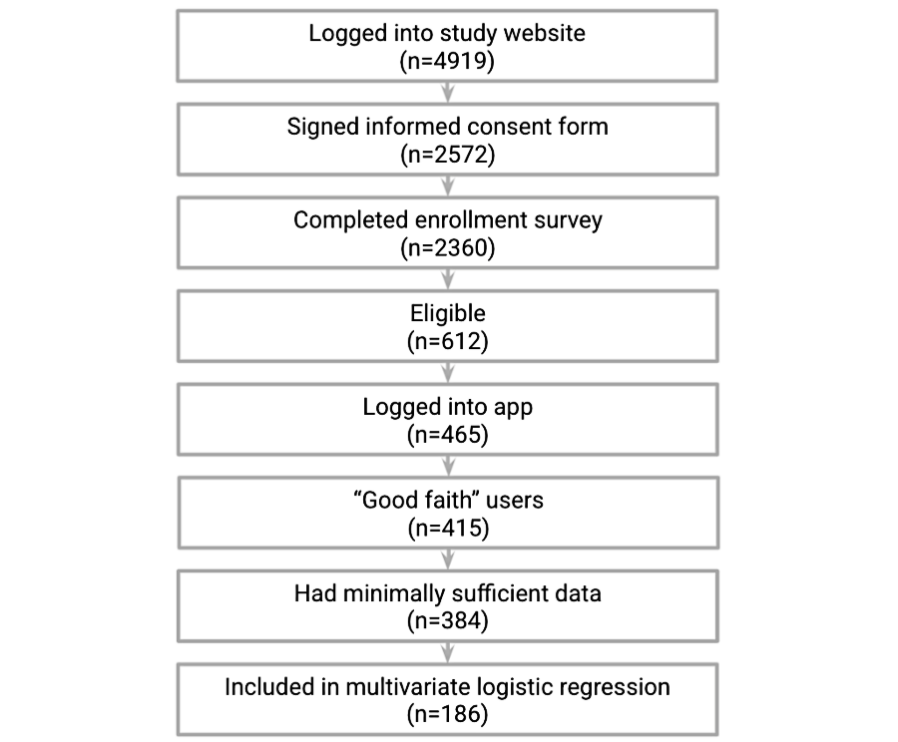
Participant flow diagram.

Enrollment started in November 2017, and the last participant finished their 12 weeks in May 2018. Figure 2 summarizes the participant drop-off at various stages. Of the 2360 participants who completed the enrollment survey, 612 (25.93%) were eligible based on the criteria reported earlier. Of those, 465 downloaded the study app, and 415 "good faith" users remained after we performed a conservative audit against potentially fraudulent activity (eg, users who seemed to be enrolled multiple times and users living in the same home as another user). The sample was further reduced to 384 users who had minimally sufficient data (ie, completed at least one in-app PHQ-9 and had at least 8 days of sufficient sensor data as per the daily filtering rule; refer to the *Methods* section).

In line with our 80:20 depressed to nondepressed recruitment ratio, the 384 participants with minimum data consisted of 313 depressed (81.5%; mean PHQ-9 at baseline 17.4, SD 4.1) and 71 nondepressed individuals (18.5%; mean PHQ-9 5.4, SD 2.9). The demographic data of these 384 participants are summarized in Table 1. Multimedia Appendix 4 provides additional demographic variables for both the 2360 individuals who completed the enrollment survey and the 384 with minimally usable data.

**Table 1:** Key demographics of the 384 participants with minimally sufficient data.

Characteristic		Participants with minimally sufficient data (n=384)	
		Depressed (n=313)	Not depressed (n=71)
**Age (years), n (%)**			
	18-29	123 (39.3)	26 (36.6)
	30-39	90 (28.8)	22 (30.9)
	40-49	56 (17.9)	14 (19.7)
	50-59	36 (11.5)	8 (11.2)
	60-69	7 (2.2)	0 (0)
	70-79	1 (0.3)	1 (1.4)
**Race, n (%)**			
	American Indian or Alaska Native	12 (3.8)	1 (1.4)
	Asian	5 (1.6)	4 (5.6)
	Black or African American	29 (9.3)	12 (16.9)
	Native Hawaiian or other Pacific Islander	2 (0.6)	1 (1.4)
	Other	14 (4.5)	1 (1.4)
	White	251 (80.2)	52 (73.2)
**Sex at birth, n (%)**			
	Female	285 (91.1)	51 (71.8)
	Male	28 (8.9)	20 (28.2)

### Data Quality

#### Survey Data Availability

From the enrolled, good faith participants (n=415), we received 83.35% (4151/4980) of all possible weekly PHQ-9 surveys over the course of the 12-week study. Analyses regarding the effects of different compensation cohorts on PHQ-9 adherence revealed no statistically significant effect of compensation group (Multimedia Appendix 1).

#### Sensor Data Availability

No data could be collected if the phone was turned off; the user force-closed the study app; the app crashed unexpectedly; or if the user revoked the persistent notification permission for the study app, as Android will eventually remove background services; in this case, data collection may resume if the user navigates back into the app. When no data were collected, it was impossible to determine the cause of data missingness from the possibilities listed earlier.

Data from specific sensors may be missing if the user permanently or temporarily revoked permission for those sensors that need specific permissions (ie, Android activity recognition, app usage, Google Fit activity recognition, location (GPS), call logs, microphone for audio diaries—not needed for ambient audio, not used for phone calls as only metadata were recorded, and text logs). A later version of the study app included a sensor that records permission status for these, but for this study, it was not possible to know whether sensor data were missing because of revoked permissions or other reasons.

After applying the daily and weekly filtering rules (refer to the *Methods* section), we had available a quality control–positive data set of 3779 participant-weeks on 384 participants (Figure 2).

**Figure 3:**
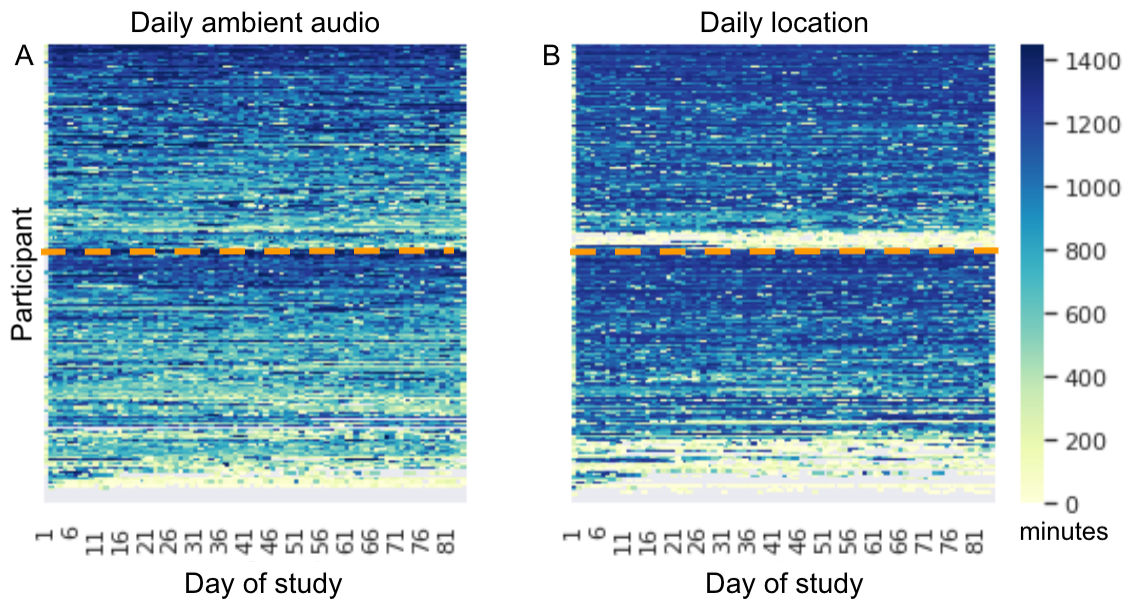
Heat maps of daily data availability. For ambient audio (A) and location (B) sensors, the color scale denotes the number of data samples on a particular participant day (between 0 and 1440, ie, the number of minutes in a day) across the 415 good faith participants (heat map rows). Gray indicates that the day was excluded altogether as per daily filtering rules. Participants are ordered in the same way in both panels, and participants that fall below the horizontal dotted line were not included in the multivariate analysis because of missingness in these or other features.

To visually characterize the patterns of missingness that result in removed days and weeks in the filtering rules, we consider for illustration purposes the daily availability of the minutely sampled sensors of ambient audio and location (Figure 3). These two sensors yielded some features with significant univariate correlations (Table 2) and were therefore used as examples. It is apparent that data missingness varies greatly among participants and their phone types (a detailed description of the phone types present in this study is presented in Multimedia Appendix 5). For example, the ambient audio sensor data are missing entirely for certain participants (see the bottom gray rows in the daily ambient audio panel in Figure 3) possibly because the hardware sensor is not present in certain phone types. Similarly, although location (GPS) capabilities are available on most phones, a special permission must be given to the app to record this sensor. The daily location panel shows that there are some participants who never have any location data (bottom gray rows) and many participants with intermittently missing location data. It is also apparent that, even on days where some data were available, the number of sampling points varies considerably on a day-to-day basis (yellow to blue gradient). We note that there are many participants whose ambient audio data or location are sufficient for analysis, but because of *other* missing features, they are excluded from the multivariate analysis (ie, they are among those participants below the horizontal dotted lines in Figure 3), as we required complete case data (refer to the *Methods* section). However, for the univariate correlation analysis, data from those participants were included if they met the daily and weekly sufficiency criteria.

**Table 2:** Univariate correlations between 34 features and Patient Health Questionnaire-9 score ordered by absolute Spearman rank correlation coefficient^a^.

Correlation with Patient Health Questionnaire-9 sum	Spearman *r*	Spearman *P* value	Benjamini-Hochberg-adjusted *P* value	Participant-weeks (n=3779), n (%)	Participants (n=384), n (%)
Voice diary sentiment	−0.26	<.001	<.001	2992 (79.2)	376 (97.9)
Reported sleep duration	−0.12	<.001	<.001	2797 (74)	308 (80.2)
Ambient audio level	0.10	<.001	<.001	3618 (95.7)	368 (95.8)
Phone call ringing until missed minutes	0.10	<.001	<.001	3334 (88.2)	360 (93.8)
Unique location clusters	−0.09	<.001	<.001	3623 (95.9)	379 (98.7)
Voice diary words per minute	−0.09	<.001	<.001	2992 (79.2)	376 (97.9)
Voice diary duration	0.08	<.001	<.001	3145 (83.2)	377 (98.2)
Battery percentage	−0.07	<.001	<.001	3778 (99.9)	384 (100)
Text message emoji count	−0.07	<.001	<.001	3460 (91.6)	366 (95.3)
Phone call count	0.07	<.001	<.001	3534 (93.5)	370 (96.4)
Voice diary pauses duration	0.06	<.001	.001	3038 (80.4)	377 (98.2)
Location entropy	−0.06	<.001	<.001	3623 (95.9)	379 (98.7)
Ambient light level	−0.06	.001	.003	3122 (82.6)	325 (84.6)
Outgoing text message sentiment score	−0.06	.001	.003	3140 (83.1)	351 (91.4)
Location variance	−0.06	<.001	.002	3623 (95.9)	379 (98.7)
Phone screen on minutes	0.05	.002	.003	3776 (99.9)	384 (100)
Audio system volume	−0.05	.002	.003	3779 (100)	384 (100)
Charging minutes	0.04	.009	.02	3773 (99.8)	384 (100)
Social apps usage	−0.04	.02	.03	3346 (88.5)	357 (93)
Time spent at home	0.04	.03	.049	3623 (95.9)	379 (98.7)
App usage missing	−0.03	.047	.08	3779 (100)	384 (100)
Outgoing phone call duration	−0.03	.08	.12	3295 (87.2)	355 (92.4)
Wellness apps usage	0.03	.08	.12	3346 (88.5)	357 (93)
Time spent at hospital	−0.03	.09	.12	3623 (95.9)	379 (98.7)
Number of Wi-Fi networks	−0.03	.08	.12	3778 (99.9)	384 (100)
Reported sleep duration missing	−0.03	.12	.16	3779 (100)	384 (100)
Communication apps usage	0.02	.24	.30	3346 (88.5)	357 (93)
Text message body size	0.02	.26	.31	3460 (91.6)	366 (95.3)
Ring volume	0.02	.32	.37	3779 (100)	384 (100)
Physically active minutes	−0.01	.38	.43	3612 (95.6)	382 (99.5)
Audio notification volume	−0.01	.42	.46	3779 (100)	384 (100)
Text message count	0.01	.66	.71	3460 (91.6)	366 (95.3)
Nearby Wi-Fi networks count	0.01	.76	.78	3364 (89)	378 (98.4)
Incoming phone call duration	0.00	.97	.97	3217 (85.1)	354 (92.2)

^a^The univariate correlations here are typically based on a subset of the filtered data set depending on the pairwise availability of the feature of interest and the weekly Patient Health Questionnaire-9 score, with data count as noted in the last two columns.

### Behavioral and Clinical Modeling

#### Case Study: Location-Derived Behavioral Features

**Figure 4:**
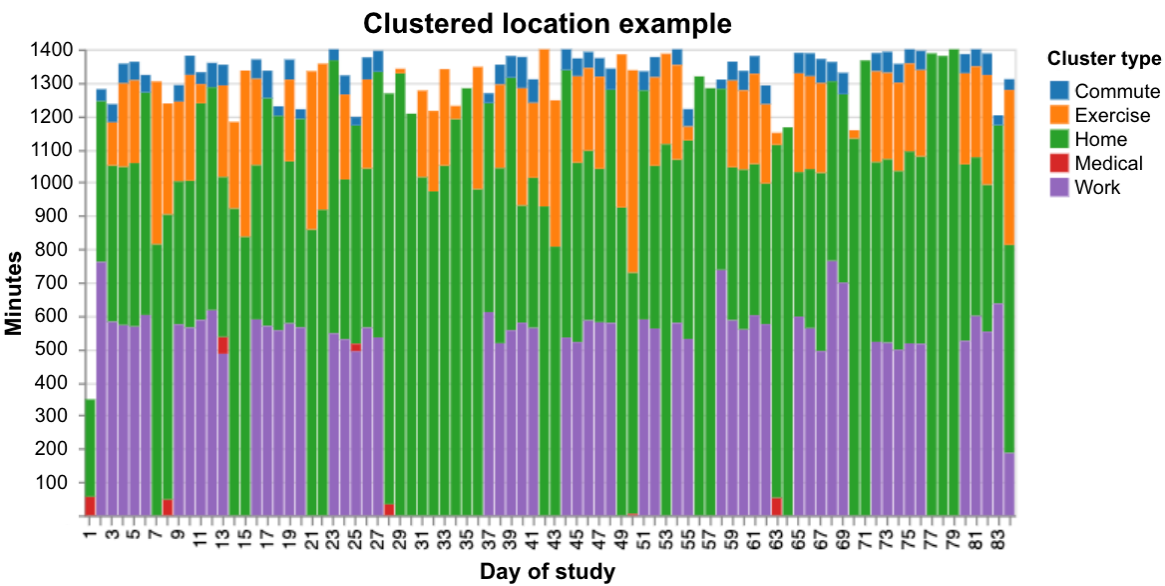
Example of clustered location data for 1 participant for the duration of the study. The total number of minutes (vertical axis) with categorized locations (denoted by various colors in the legend) are plotted as stacked bars for each day of the study on the horizontal axis. Note the week-long increased homestay starting on day 28.

To anecdotally illustrate the quality of insights into real-world patient behavior that can be obtained from passive sensors, Figure 4 depicts a graphical summary of 1 participant’s daily clustered location data. It is apparent that this participant had a strong weekly routine, going to work for approximately 550 minutes (approximately 9 hours) for 5 days, followed by no time spent at work for 2 days. The participant also regularly spends time at exercise locations (eg, a gym or a sports center) and has some medical appointments. An interesting disruption in this routine can be observed starting on day 28 of the study, during which the participant had a medical appointment, followed by 8 days without any time at work or commuting.

#### Univariate Correlations Analysis With PHQ-9

To move beyond anecdotal evidence that smartphone sensors may be clinically relevant, we started with a simple question asking which, if any, behavioral features show correlations with the PHQ-9. To this end, we considered the 3779 participant-weeks (across 384 participants) available after filtering. Among the 34 behavioral features selected for analysis (refer to the *Methods* section), 11 showed a significant (*P*<.001) Benjamini-Hochberg-adjusted correlation with weekly PHQ-9 (Table 2). Visualizations of the spread of the PHQ-9 values and the associations between the significant behavioral features and the PHQ-9 values can be found in Multimedia Appendix 6.

In descending order of the absolute strength of the relationship, we found that a more negative sentiment of the voice diary—a measure derived from a sentiment classification algorithm [21] returning measures from −1 (extremely negative) to 1 (extremely positive)—is associated with a higher PHQ-9 score. Self-reported sleep was negatively correlated, meaning that the less sleep the participant reported that week, the higher their PHQ-9 score was. A higher ambient audio level, that is, more noise registered by the phone, was associated with higher depression severity. Letting the phone ring for longer periods until the call was missed was also associated with higher depression severity. Unique location clusters or how many different places (eg, home, work, or unlabeled clusters) the participant visited that week was negatively correlated with PHQ-9, meaning that the fewer different locations visited in a given week, the higher the PHQ-9 score. Two other measures from the voice diary, words spoken per minute (speaking rate) and the recorded duration of the diary, also showed a significant relationship: the fewer words spoken per minute and the longer the duration of voice diary, the higher the PHQ-9. The battery percentage (between 0% and 100%) was negatively associated, meaning that if the weekly mean battery percentage was lower, PHQ-9 was higher. The number of emojis in outgoing text messages showed a negative correlation, that is, a lower number of emojis was associated with a higher PHQ-9 score. Receiving or making more phone calls per week was associated with higher PHQ-9 scores. Finally, location entropy—measuring the variability in how much time a participant spent at different location clusters—showed a negative association: the less variability in where participants spent time, the higher the PHQ-9.

#### Multivariate Logistic Regression

**Figure 5:**
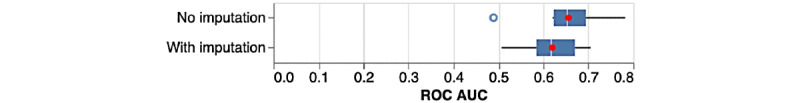
Box plots of areas under the curve from 10-fold cross-validation of logistic regression predicting depression status, for the nonimputed and imputed data sets. The white line represents the median, and the red dot represents the mean. AUC: area under the curve; ROC: receiver operating characteristic.

Recall that, after filtering, there were 3779 participant-weeks available for 384 participants. Of those, 1013 weeks from 186 participants were complete cases for the 34 features and were therefore included in an initial multivariate analysis (the correlation matrix of the 34 features is presented in Multimedia Appendix 7). For these participant-weeks, 60.91% (617/1013) were classified as "depressed" (PHQ-9≥10), whereas 39.09% (396/1013) were "nondepressed" weeks (PHQ-9 score <10). We used an elastic net penalized multivariate logistic regression for predicting weekly depression status and evaluated performance with the receiver operating characteristic AUC of 10 cross-validated folds. The mean AUC of the 10 folds was 0.656 (SD 0.079; Figure 5).

We also attempted to recover some participant-weeks that were dropped for not being complete cases by performing within-participant mean imputation. This resulted in a data set of 2016 participant-weeks for 197 participants, with 1173 (58.18%) depressed and 843 (41.82%) nondepressed weeks. Some weeks could not be recovered because a feature was missing for all 12 weeks and could therefore not be imputed using this simple within-participant procedure. With a mean AUC of 0.620 (SD 0.062; Figure 5), models trained using these imputed data performed worse on average than the models trained without imputed data, although there was less spread in the 10 AUC values.

## Discussion

### Principal Findings

The results of this feasibility study showed that it is possible to remotely engage a depressed study population quickly and with high adherence (>80%), data collection from a broad range of Android smartphone sensors is possible, and multiple features from multiple feature families can be extracted. We further provided a proof-of-concept demonstration of multivariate modeling of depression status.

It is also clear that passive sensor data collection using the "bring-your-own-device" approach is complex, especially across the diverse ecosystem of Android devices (Multimedia Appendix 5). The data exhibit high levels of missingness and noise related to the characteristics of the phone and the user. Furthermore, our choice of minimally required daily and weekly data amounts was arbitrary. Future studies on a scaled-up sample should develop more sophisticated models of missingness imputation that account for differences in hardware sensors and phone type quality. Furthermore, a large-scale sample would allow for more complex (eg, nonlinear) modeling of clinical outcomes, particularly considering interactions with demographics and other covariates. Therefore, to interpret this report, it is important to bear in mind that this sample skews heavily female compared with the population of individuals with depression in the United States. It also only consists of individuals who own Android phones (refer to the *Limitations* section).

Univariate correlations of 34 behavioral features showed promising correlations with the clinically validated PHQ-9 scores. Some of those replicate previous findings on similar features, for example, the negative associations between location entropy and location variance with PHQ-9 [22], the positive relationship between the duration of a diary and PHQ-9, and the negative relationship between words per minute and PHQ-9 (compare the findings herein with the total recording length [*r*=0.20] and speaking rate as syllables per second [*r*=−0.23] association with the Hamilton Depression Rating Scale in Mundt et al [23]). Of note, the univariate correlation coefficients reported in our study are, in many cases, smaller than those reported for similar behavioral features in other studies despite our larger sample (refer to the systematic review by Rohani et al [15], specifically the Multimedia Appendix 5 for a summary of the correlation coefficients from studies on patients with unipolar and bipolar depression). We posit that these inconsistencies might be because of the winner’s curse—a statistical phenomenon discussed often in genome-wide association studies [24]—where associations that are close to a decision threshold are more likely to be an overestimation of the true association in the generating sample, leading to poorer replication in a separate sample (refer to the paper by Pratap et al [16] for a larger scale replication study that found no association for phone usage and a small association for GPS mobility). We also hypothesize that, for the prediction of mood scores, it may be more beneficial to define participant-level models focusing on changes against oneself over time rather than population-level models focusing on deviations from the population mean [16].

Another aim of this study was to demonstrate the potential clinical utility of behavioral features derived from passive sensors to help inform clinical practice. The location example (Figure 3) shows one area in which a clinician could gain insights about a patient’s routine that could augment clinical decision-making and strengthen feedback-informed care [25]. This and other passive features such as physical activity minutes, social app usage, or how noisy a patient’s environment is could perhaps give clinicians a more holistic and continuous picture of their patient’s circumstances than would likely be available from momentary in-clinic visits alone. However, although the statistically significant correlations suggest some clinical relevance, future validation work is needed to establish whether some of the behavioral features alone or a multivariate model provide a sufficiently reliable signal to warrant clinical actionability.

Notably, active tasks such as voice diaries yielded rich insights into the patient’s previous week, resulting in the strongest correlation with the PHQ-9 (*r*=0.26). Active tasks collected via a mobile app, be it voice diaries or even standard clinical measures, such as the PHQ-9, might offer another avenue for gathering perhaps not continuous but more frequent data points about a patient’s well-being. As they are completed without any burden on the clinician, such self-administered measurements could remove some of the barriers to the adoption of measurement-based care in psychiatry [7]. Furthermore, one could imagine the development of an early alert system or a case ranking system based on these digitally deployed measurements that could draw attention to deteriorating patients without them having to appear in person and without them having to directly tell their therapist the uncomfortable fact that they are not getting better [25,26].

For the active measurements in particular, we believe that this study is an encouraging example of how an intuitive, fun, and engaging user interface can make interacting with an app around a generally difficult topic, such as depression, not only less burdensome but perhaps even pleasant and beneficial. In fact, anecdotal feedback about using our app suggests as much. One patient reported “[t]he app made me feel like I had an everyday purpose. I looked forward to filling it out. I enjoyed the interaction.” Another patient stated “[i]t put my mental health into perspective and I had to answer how I was feeling, not what people expect me to feel.”

### Limitations

The entirely open recruitment through advertisements in this study led to a sample that was different from the larger population of individuals with depression in the United States (compare the national survey data in Luciano and Meara [27]). Most notably, our depressed sample consisted of more females (285/313, 91.1% vs 12,182/20,313, 59.97% in the national survey [27]). Furthermore, enrollment was restricted to participants owning phones with an Android operating system. All these factors lead to limited generalizability of the findings. Future recruitment strategies should involve more targeted advertising or partnering with companies or health care systems to recruit a more representative sample. We also enrolled participants based on their self-report of symptoms and treatment history on good faith; however, future studies with strengthened clinical verification methods (eg, concurrent donation of clinical records or prescription records to the research study) may enhance the validity of clinical measures.

PHQ-9 adherence (>80%) was found to be the same for all three compensation cohorts (Multimedia Appendix 1), but future work is needed to test whether adherence with a similarly engaging app but less compensation than the lowest level (US $135) will lead to similarly high adherence levels.

The semantic location features, especially locations marked as "exercise", yielded an unexpectedly high number of false positives upon manual inspection. Thus, future validation studies are required to improve these semantic location results.

Passive sensor collection, as carried out in this study, requires the user to agree to the collection of large amounts of personal data, and it is unclear whether patients outside a research context would be willing to share these data. A mitigation for this issue could be federated learning, a technique to train machine learning models locally on devices without having to pool them in a central storage location.

Despite these limitations, this study has an important place in the field with regard to the potential of low-burden collection of surveys as well as smartphone features in detecting changes in depression over time. This information could be used to improve the monitoring of treatment success or enhance the selection of treatments for each patient.

## Appendix

Multimedia Appendix 1 Compensation cohort analyses.

Multimedia Appendix 2 Informed consent form.

Multimedia Appendix 3 User experience research and user-centered design.

Multimedia Appendix 4 Study population characteristics.

Multimedia Appendix 5 Phone types.

Multimedia Appendix 6 Patient Health Questionnaire-9 figures.

Multimedia Appendix 7 Feature correlation matrix.

## Abbreviations

AUC: area under the curve
DALY: disability-adjusted life year
DBSCAN: density-based spatial clustering of applications with noise
ICF: informed consent form
PHQ: Patient Health Questionnaire
YLD: year lived with disability
