# Supplementary material for: Toward a Mobile Platform for Real-world Digital Measurement of Depression: User-Centered Design, Data Quality, and Behavioral and Clinical Modeling
Source: JMIR Ment Health. 2021 Aug 10;8(8):e27589. doi: 10.2196/27589 (PMC8386379; doi:10.2196/27589)
Supplement: Multimedia Appendix 1 [file mental_v8i8e27589_app1.pdf]

Compensation cohort analyses

Effects of compensation on PHQ-9 adherence

To test whether compensation affected the number of surveys completed, participants were randomly divided into 6 test groups. The total maximum study compensation was either $135, $265, or $530, with each of those subsets divided into two, with weekly PHQ-9 completion being compensated at either $3 or $4, $6 or $8, and $12 or $16, respectively. The rest of the compensation was proportionally assigned upon completing other surveys, including weekly audio diary recordings, weekly quality of life surveys, daily sleep and wake times and sleep quality surveys, and daily mood surveys.

Methods: Generalized Estimating Equations (GEE)

In order to determine whether time (continuous, one measure every week for 12 weeks) and cohorts of maximal possible payments (3 levels: $135, $265, or $530) had an influence on whether the PHQ-9 survey was completed (binary response), we defined a Generalized Estimating Equations (GEE) model with all possible interactions using the `statsmodels.formula.api.gee` Python package, specifying the family as Binomial and the covariance structure as Autoregressive (AR1).

Results

By maximum compensation

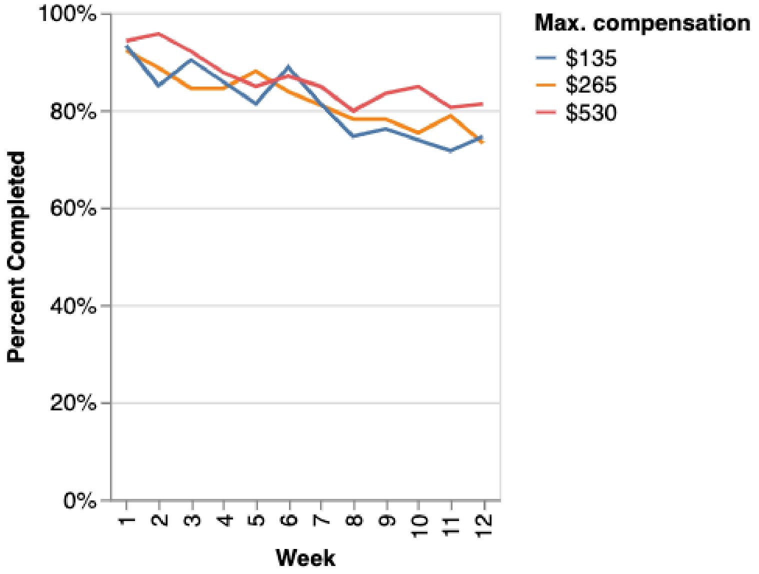

Figure S1: Percentage of completed PHQ-9 questionnaires over the course of the 12 week study, stratified by compensation cohort.

From the enrolled, good faith participants (n=415; see Figure 2), we received 83% of all possible weekly PHQ-9 surveys over the course of the 12 week study. To determine whether adherence differed by maximum possible compensation ($135, $265, or $530), we conducted a GEE analysis (Table S2). We found a significant main effect of week (P<0.001, odds ratio=0.89), indicating that adherence decreased over the course of the study. However, there are no main effects or interactions involving total compensation, meaning that this reduction of adherence over time was the same for all compensation levels (as graphically depicted in Figure S1). These results remained the same using an unstructured covariance structure.

Table S1: Results of the GEE modelling of PHQ-9 adherence for all good faith participants (n=415) using AR-1 covariance structure. Total compensation is treatment-coded against $135 as the reference category. Significant values are highlighted with asterisks in this and all following tables (*: <.05, **: <.01, ***: <.001).

	Log Odds	Std Err	z	P>|z|	Odds ratio
Intercept	2.346	0.24	9.99	<.001***	10.44
total compensation[$265]	0.011	0.33	0.04	0.97	1.01
total compensation[$530]	0.264	0.33	0.81	0.42	1.30
week	-0.115	0.02	-4.83	<.001***	0.89
week:total compensation[$265]	0.001	0.03	0.02	0.99	1.00
week:total compensation[$530]	0.013	0.03	0.39	0.70	1.01

By maximum compensation and depression status

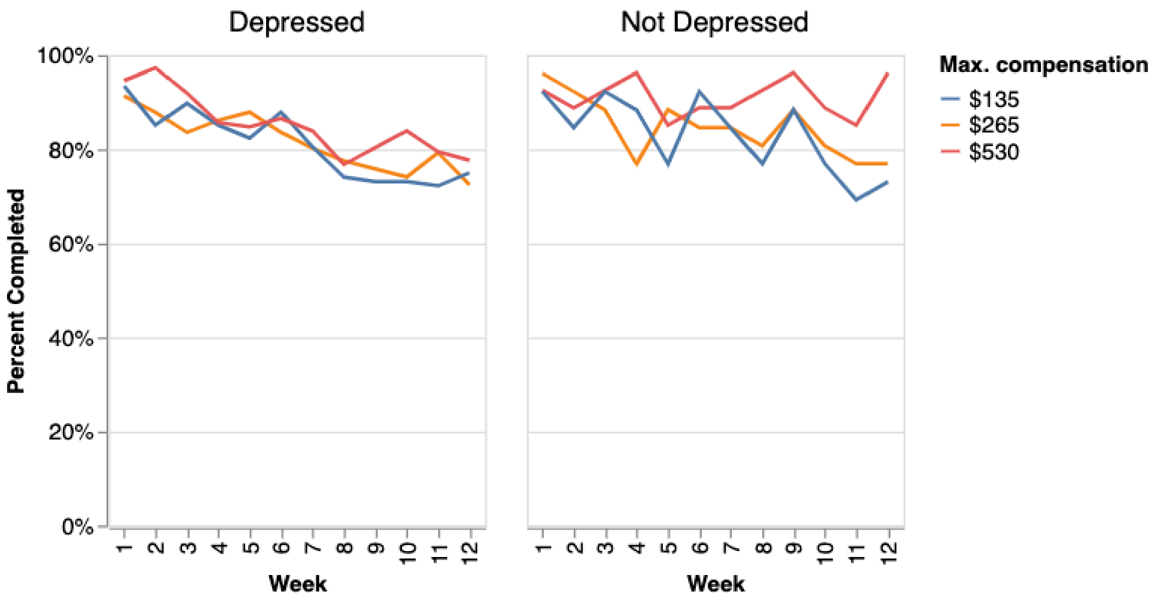

Figure S2: Percentage of completed PHQ-9 questionnaires over the course of the 12 week study by compensation cohort and depression status at baseline (Depressed: PHQ-9 >= 10, Non-Depressed: PHQ-9 < 10).

In order to determine whether time (continuous, one measure every week for 12 weeks), payment cohorts (3 levels, $135, $265, or $530), and depression status at baseline (2 levels: depressed and not depressed) had an influence on whether the PHQ-9 survey was completed (binary response, graphically depicted in Figure S2), we defined a Generalized Estimating Equations (GEE) model with all possible interactions (Table S3) using the `statsmodels.formula.api.gee` Python package, specifying the family as Binomial and the covariance structure as Autoregressive (AR1). Note that there were 4,032 participant-weeks from 336 participants that were not depressed at baseline.

We found again a significant main effect of week ($P < 0.01$, odds ratio = 0.89). However, there were no main effects or interaction effects involving compensation cohort (“comp”) or depression status (“cohort”), except for the weakly significant week:cohort[Non-Dep]:comp[530] interaction ($P = 0.045$, odds ratio=1.18) which reflects higher adherence over the 12 weeks for non-depressed participants who would receive $530 (red line in right panel of Figure S2) compared to the reference group, namely depressed participants who could reach $135 (blue line in left panel). Importantly, this interaction was no longer significant ($P = 0.12$) when using an unstructured covariance structure, but none of the other terms changed significance.

Table S2: Results of the GEE modelling of PHQ-9 adherence by maximum compensation cohort and depression status for all good faith participants (n=415) using AR-1 covariance

structure. Comp is treatment-coded against $135 as the reference category, cohort is treatment-coded against Depressed participants.

	Log Odds	Std Err	z	P>|z|	Odds ratio
Intercept	2.317	0.25	9.13	<.001***	10.14
comp[$265]	-0.016	0.35	-0.05	0.96	0.98
comp[$530]	0.350	0.35	1.00	0.32	1.42
cohort[Not Dep.]	0.165	0.66	0.25	0.80	1.18
comp[$265]:cohort[Not Dep.]	0.174	0.95	0.18	0.85	1.19
comp[$530]:cohort[Not Dep.]	-0.541	0.90	-0.60	0.55	0.58
week	-0.113	0.03	-4.26	<.001***	0.89
week:comp[$265]	0.000	0.04	0.00	1.00	1.00
week:comp[$530]	-0.011	0.04	-0.28	0.78	0.99
week:cohort[Not Dep.]	-0.013	0.06	-0.20	0.84	0.99
week:comp[$265]:cohort[Not Dep.]	0.002	0.08	0.03	0.98	1.00
week:comp[$530]:cohort[Not Dep.]	0.164	0.08	2.00	0.045*	1.18

By PHQ-9 payment

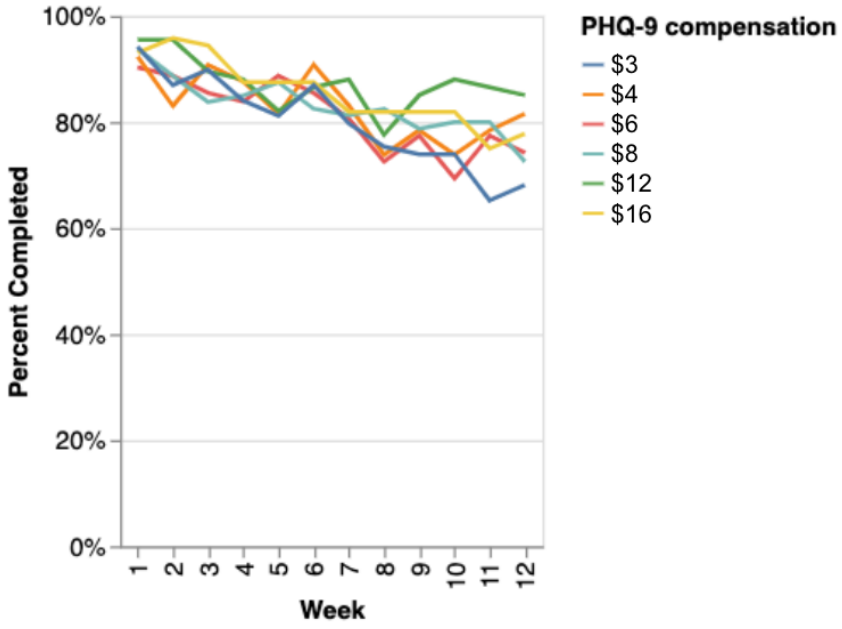

Figure S3: Percentage of completed PHQ-9 questionnaires over the course of the 12 week study by PHQ-9 compensation.

A final GEE analysis was conducted to determine whether the 6 levels of PHQ-9 compensation ($3 vs. $4, together with the other surveys adding up to $135, $6 vs. $8 adding up to maximally $265, and $12 vs. $16 adding up to maximally $530, Figure S3) led to differences in PHQ-9 adherence (Table S4). We find again an effect of week (p<0.01, odds ratio=0.86), but no other terms emerge as significant.

Table S3: Results of the GEE modelling of PHQ-9 adherence by PHQ-9 payment for all good faith participants (n=415) using AR-1 covariance structure. PHQ-9 comp is treatment-coded against $3 as the reference category.

	Log Odds	Std Err	z	P>|z|	Odds ratio
Intercept	2.499	0.34	7.26	<.001***	12.17
PHQ-9 comp[$4]	-0.312	0.47	-0.66	0.51	0.73
PHQ-9 comp[$6]	-0.305	0.49	-0.63	0.53	0.74
PHQ-9 comp[$8]	-0.003	0.46	-0.01	1.00	1.00
PHQ-9 comp[$12]	0.072	0.48	0.15	0.88	1.07
PHQ-9 comp[$16]	0.155	0.45	0.34	0.73	1.17
week	-0.151	0.03	-4.62	<.001***	0.86
week:PHQ-9 comp[$4]	0.077	0.05	1.60	0.11	1.08
week:PHQ-9 comp[$6]	0.050	0.05	1.05	0.29	1.05
week:PHQ-9 comp[$8]	0.025	0.04	0.58	0.56	1.03
week:PHQ-9 comp[$12]	0.072	0.04	1.64	0.10	1.07
week:PHQ-9 comp[$16]	0.029	0.05	0.60	0.55	1.03

Conclusion on Compensation Levels

PHQ-9 adherence was found to be unaffected by compensation cohort in this study, both in terms of maximum compensation ($135, $265, or $530) and in terms of PHQ-9 compensation ($3, $4, $6, $8, $12, or $16). Depression status showed only a weak effect (P = 0.045), when comparing the lowest compensation group in the depressed cohort to the highest compensation group in the non-depressed cohort. Taken together, these effects suggest that compensation level had at most only a very minimal impact on the PHQ-9 adherence in this selected sample (see Limitations in main text for details on issues of generalizability to more representative samples).

Effects of compensation on sensor data availability

To test whether the random assignment to the three compensation cohorts affected the amount of sensor data received we conducted a GEE analysis for the two example sensors used in the main text: ambient audio sensor and location sensor.

Ambient audio data availability by compensation cohort

Figure S4 shows, stratified by the three compensation cohorts, the average number of daily ambient audio samples received over the course of the 84-day study. It is apparent that there is little systematic variation across the compensation cohorts, however, less data is received on day 1 and day 84.

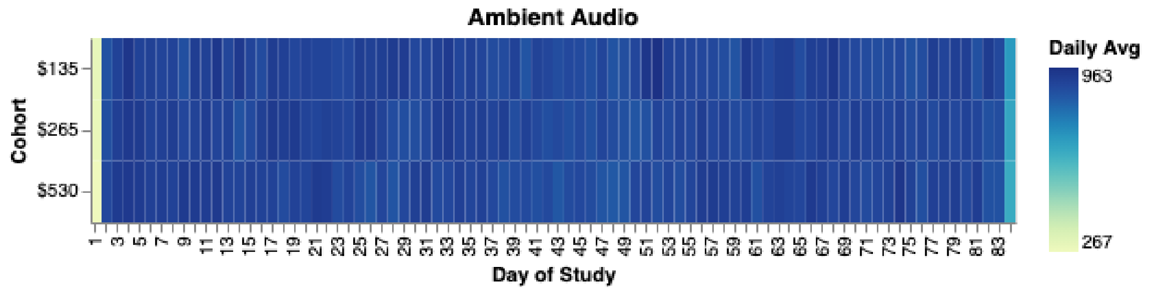

Figure S4: Daily average count of samples recorded from the ambient audio sensor, stratified by the three different compensation cohorts.

In order to statistically determine whether time (continuous, one data point per day for 84 days) and cohorts of maximal possible payments (3 levels: $135, $265, or $530) had an influence on the number of samples received (count data of maximally 1440 minutes per day), we defined a GEE model with all possible interactions, specifying the family as Poisson and the covariance structure as Exchangeable (also called “compound symmetry”).

The model yielded no significant results other than the intercept, suggesting that the compensation cohort did not affect the amount of ambient audio sensor data received (see Table S5).

Table S4: Results of the GEE modelling of ambient audio sensor availability for all good faith participants (n=415). Total compensation is treatment-coded against $135 as the reference category.

	Coefficient	Std Err	z	P>|z|
Intercept	6.795	0.03	256.76	<.001***
total compensation[$265]	0.013	0.04	0.34	.73
total compensation[$530]	0.014	0.04	0.35	.73
day of study	0.000	0.00	-0.98	.33
day of study:total compensation[$265]	0.000	0.00	-0.10	.92
day of study:total compensation[$530]	0.000	0.00	-0.26	.80

Location data availability by compensation cohort

Figure S5 shows the average number of daily location samples received, separately for the three compensation cohorts. As with the ambient audio samples, there appears to be little systematic variation across the compensation cohorts.

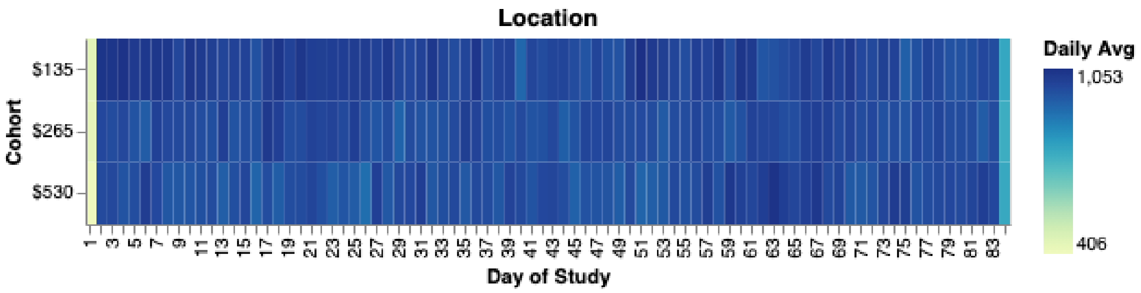

Figure S5: Daily average count of samples recorded from the ambient audio sensor, stratified by the three different compensation cohorts.

We specified the same GEE model as for ambient audio counts to statistically determine whether the compensation cohort influenced location data availability. Again, the model yielded no significant results other than the intercept, suggesting that compensation cohort similarly did not affect the amount of location sensor data received (see Table S6).

Table S5: Results of the GEE modelling of location sensor availability for all good faith participants (n=415). Total compensation is treatment-coded against $135 as the reference category.

	**Coefficient**	**Std Err**	**z**	**P>|z|**
Intercept	6.850	0.03	241.95	<.001***
total compensation[$265]	-0.050	0.04	-1.17	.24
total compensation[$530]	-0.061	0.05	-1.32	.19
day of study	0.000	0.00	-0.56	.58
day of study:total compensation[$265]	0.000	0.00	0.95	.34
day of study:total compensation[$530]	0.001	0.00	1.56	.12
