# Supplementary material for: Toward a Mobile Platform for Real-world Digital Measurement of Depression: User-Centered Design, Data Quality, and Behavioral and Clinical Modeling
Source: JMIR Ment Health. 2021 Aug 10;8(8):e27589. doi: 10.2196/27589 (PMC8386379; doi:10.2196/27589)
Supplement: Multimedia Appendix 2 [file mental_v8i8e27589_app2.pdf]

Informed Consent Form (ICF)

RESEARCH SUBJECT INFORMATION AND CONSENT FORM

**TITLE:**	The Project Baseline Mood Study ("Mood Study")
**PROTOCOL NO.:**	2017-MOOD-001<br>WIRB® Protocol #20172351
**SPONSOR:**	Verily Life Sciences LLC
**INVESTIGATOR:**	Honor Hsin, MD PhD<br>269 East Grand Avenue<br>South San Francisco, California 94080<br>United States
**STUDY-RELATED PHONE NUMBER(S):**	The Mood Study User Support Center<br>(844) 641-8390<br>mood-study@projectbaseline.com

A person who takes part in a research study is called a participant. In this consent form "you" always refers to the participant.

SUMMARY

You are being asked to be in a research study. The purpose of this consent form is to help you decide if you want to be in the research study.

Things to know before deciding to take part in a research study:

- The main goal of a research study is to learn things to help patients in the future.
- This research study is NOT regular medical care. The main goal of regular medical care is to help each patient get better.

Participating in this study is voluntary. If you do not wish to participate, this will not affect any current or future care or associated benefits or treatment that you receive from your doctor.

Please read this form carefully. **One important thing to know about this study is that the content of your outgoing text messages, as well as other information on your phone like your location, app usage, and phone usage will be collected by the study sponsor, a company called Verily. Detailed information on exactly what information is collected can be found in the table below.**

Additional information about this study can be found on the Mood Study website: www.projectbaseline.com/mood. You should take your time to decide whether or not you want to join this research study. You should not join this research study until all of your questions have been answered. Signing this form is equivalent to signing a paper consent form.

PURPOSE OF THE STUDY

This study has two purposes (1) to discover the best way to collect information about mental health from smartphones using a mobile application called the Mood Study App and (2) to learn the best ways to engage and compensate participants in studies that collect data from smartphones. The Mood Study App is a mobile application that collects and stores information related to a user's mental health, including mood, physical activity, social function, and sleep patterns. The Mood Study App collects data through sensors in your smartphone as well as active data entry from the user, such as surveys.

The information collected in this study will not be used to diagnose medical conditions or make treatment decisions. **Participation in this study is not a substitute for your usual medical care. If you choose to take part in this study, you should continue with your usual medical care and contact your care provider(s) for any health related questions or concerns.**

This study will involve up to 1000 people nationwide. The study will enroll 800 participants with depression and 200 participants without depression.

All study procedures will be conducted remotely through your personal Android smartphone. You will need to download the Mood Study App to your phone in order to participate in this study. These tasks will be described in the Procedure Section.

STUDY ELIGIBILITY

In order to participate in this study, you will need to meet certain eligibility criteria.

You are eligible if you are:

- Age 19 years or older.
- A U.S. resident.
- Able to read and speak English.
- Able and willing to provide informed consent.
- The owner and sole user of an Android smartphone with a data plan.
- Able to download the Mood Study App from the Play Store onto your Android phone.
- Agree to provide a phone number and email address to enable feedback through communication.
- Agree to provide a mailing address to receive compensation for being in the study.

This study will recruit participants with depression and without depression. Participants with depression are eligible if they have started treatment for depression under the care of a licensed clinician (medications and/or talk therapy) within 2 weeks prior to study entry.

You are not eligible if you:

- Are a current Alphabet Inc. or affiliated company (including Verily) employee, temporary worker, vendor, or contractor or immediate family member of one
- Have active suicidal thoughts.

PROCEDURES
If you agree to take part in this study, you will be asked to sign this consent form. A copy of the signed form will be sent to you by email.

Mood Study Profile
After you sign the consent form, you will be asked to create a Study Profile and provide information about yourself to make sure you are eligible to be in this study. Your Study Profile is a collection of information about you and your health. You will be asked to provide the following types of information:

- Contact information, which may include your phone number, email, and current home address
- Demographics information, including information about your age, race, and gender
- Information about your mental health, including whether or not you have previously or are currently receiving treatment for depression and what type(s) of treatment you have received

You will also be asked to complete a brief questionnaire about your mental health, called the Patient Health Questionnaire-9 (PHQ-9).

Your Mood Study Profile will be used to:
- Decide if you are eligible for the Mood Study
- Understand characteristics of eligible and non-eligible individuals
- Notify you of future studies that you might be interested in

Your Study Profile may continue to be used for these purposes even if you are not eligible for the Mood Study.

The study team will use a computer system to determine if you qualify for the Mood Study. If you are eligible to participate in the study, you will be notified of your eligibility by the study team. If you choose to continue in the study, here is what will happen next:

Baseline Assessments
You will be asked to complete the following surveys and assessments through the Mood Study website listed in the Summary Section using your mobile phone or other electronic device, such as a desktop or laptop computer.
- Information about your overall medical history, including mental health history
- Additional demographics information, such as your occupation, marital status, and education, and salary range
- Assessments about your mental health, emotional support, and friendships
- Questionnaires about anxiety, drug and alcohol use, and post-traumatic stress disorder (PTSD)

Mobile Application Download
After you complete all baseline assessments, you'll be instructed on how to download the Mood Study App to your Android smartphone. Before installing the Mood Study App, it is strongly recommended that you set a screen lock to help secure your Android phone. It is important that you are the sole user of the phone, as you will need to download and log into the Mood Study App using the same Google account associated with the Android smartphone. If you need help with downloading or using the Mood Study App, you will be able to request that a User Support

Center team member contact you through your email address or phone number. You will use the app to complete all future assessments.

After you complete your baseline assessments and download the Mood Study App, you will use the app every day and complete the below described tasks over the following 12 weeks. You may receive reminders to complete tasks via email, phone calls, text message, or in app reminders.

The Mood Study App will collect information about you from your phone that is described in the table below.

Mood Study App Use (Weeks 1 to 12)

Daily Activities
You will be asked to rate your physical activity level from the previous day and report your wake time and bedtime. These tasks will take approximately 1 minute or less to complete.

You will be asked to rate your mood and effort required to complete daily chores / duties. You will also be asked questions related to your sleep. These tasks will take approximately 2 minutes or less to complete.

Weekly Activities
Each week you will be asked to complete two questionnaires about your mental health and its impact on your daily life.

You will also be asked to report any changes to how you are helping or treating your depression.

Finally, you will be asked to provide an audio recording of yourself speaking for at least 30 seconds.

These weekly questionnaires and voice log will take, in total, approximately 15 minutes or less to complete.

Monthly (at Weeks 4, 8, and 12) Activities
During weeks 4, 8, and 12, you will be asked to complete two questionnaires about your social function and life satisfaction. . These questionnaires will take approximately 15 minutes or less to complete.

At Study Completion (Week 12)
At the end of the study, you will be asked to provide your feedback on the use of the Mood Study App and your experience as a study participant. These questions will take approximately 15 minutes or less to complete.

COLLECTION OF SENSITIVE INFORMATION FROM YOUR PHONE

Throughout your participation in the study, as long as the Mood Study App is on your phone, the app will collect information from your phone and sensors on your phone. The app will collect information that was on your phone for up to one week before you started the study.
The collection of information will be ongoing while the app is installed on your phone. In other words, information will be collected even if the app is not open. The app will stop collecting information only when you uninstall it from your phone or when your phone does not

have network connectivity (for example, if it is turned off or in airplane mode). When you exit the study, you will remove the app from your phone.

The types of information which may be collected and the time of collection are detailed in the table below. The app may collect some or all of the information types listed.

Please review this table carefully:

Information Collected	Privacy Considerations	Collection Start Time
Your Text Messages:<br>We will collect information about your text messages, including the full content of outgoing texts.<br><br>For outgoing texts, we will store the content of the text message itself, in addition to summary data, including the message type, a unique identifier to represent the recipient's phone number (but not the actual number itself), and unique identifiers for each message and message thread.<br><br>For incoming texts, we will not store the content of the text message. Instead, we'll capture the following summary data: word count, character count, emoji count, emoji type, non-latin character count, and blurred text (the original message will not be known).<br><br>For incoming texts, we will also collect whether the message was read, the message ID, the message type, a non-reversible hash (ie, a unique identifier) of the other number, and the thread ID.	Phone numbers are non-reversibly hashed out so that the actual phone numbers are hidden and we don't see them.<br><br>All outgoing text messages will be captured and stored.<br><br>Incoming text messages will be masked so that the content of the original message will never be stored. Specifically, the characters of the original text message will be replaced with a string of unreadable characters and symbols.	Up to 2 days prior to the first time you log into the Mood Study app
Your Phone Calls: We collect summary data about incoming and outgoing phone calls. We do not record your calls, but keep track of when calls are made and for how long.	Phone calls are never recorded. Only summary data on phone calls is collected (start time, stop time, missed call, hashed out phone number). Phone numbers are non-reversibly hashed out so that the actual phone numbers are hidden.	After you log into the Mood Study app for the first time

Your App Use: We track which apps are running on your phone and for how long. We also measure network bandwidth usage by app. Only the names of Google apps and apps from Play Store are tracked.	Specific app names are accessible, however your interactions inside each app are not.	Up to 1 week prior to the first time you log into the Mood Study app
Your Calendar: We read your calendar events and details, including event title and description, location, time, number of invitees, and recurring vs. non-recurring.[1]	All event details are accessible, however we remove the names of people in event titles and descriptions and do not store the names of invitees. Company/institution names included in event titles and descriptions will be collected. Calendar entries that are collected by the app, but later deleted by you, will still be stored.	Up to 1 week prior to the first time you log into the Mood Study app
Location: We keep track of the estimated latitude and longitude (GPS coordinates) of your phone	It may be possible to identify your recent locations	After you log into the Mood Study app for the first time
Your Activity Level: We track your estimated activity from a predefined set of activities (eg, walking, running, biking, sitting). This data comes from the phone itself or wearable devices connected to your phone through Google Fit.	There is minimal risk that this information can be used to identify you	Up to 1 day prior to the first time you log into the Mood Study app
Light Level: Ambient light level detected by the phone	There is minimal risk that this information can be used to identify you	After you log into the Mood Study app for the first time
Pressure: Ambient air pressure measured by the phone	There is minimal risk that this information can be used to identify you	After you log into the Mood Study app for the first time
Proximity: Measurement of whether an object is within a given distance. This can be used to help determine if the phone is in a pocket or bag.	There is minimal risk that this information can be used to identify you	After you log into the Mood Study app for the first time
Step Count: Estimated number of steps taken. This data comes from the phone	There is minimal risk that this information can be used to	Up to 1 day prior to the first time you log into the

[1] Note that while calendar was consented as a sensor in the ICF (as shown above), due to technical difficulties this sensor was never implemented in the app itself, and no calendar data was recorded during the study.

itself or wearable devices connected to your phone through Google Fit.	identify you	Mood Study app
Accelerometer: Measures the tilting motion and orientation of your phone	There is minimal risk that this information can be used to identify you	After you log into the Mood Study app for the first time
Gyroscope: Adds a dimension to the information supplied by the accelerometer by tracking rotation or twist	There is minimal risk that this information can be used to identify you	After you log into the Mood Study app for the first time
Magnetometer: Used to approximate the direction your phone is pointed in (eg, North)	There is minimal risk that this information can be used to identify you	After you log into the Mood Study app for the first time
Volume: Volume settings on your phone for different sounds (eg, alarm, music, ringer).	There is minimal risk that this information can be used to identify you	After you log into the Mood Study app for the first time
Ambient Noise: Periodically samples the ambient volume (not speech content).	There is minimal risk that this information can be used to identify you. This sensor captures average sound volume readings, not speech content.<br><br>This sensor will be disabled during all phone calls or when any other app that uses the microphone is active.	After you log into the Mood Study app for the first time
Bluetooth: Bluetooth devices in the vicinity and if bluetooth is enabled on your phone	Bluetooth device names are non-reversibly hashed out so the actual bluetooth device names are hidden	After you log into the Mood Study app for the first time
Device: Information on your phone and operating system	There is minimal risk that this information can be used to identify you	After you log into the Mood Study app for the first time
Wifi Networks: Names of wifi networks in the area (not just the one connected to your phone) and if wifi is enabled on your phone	Wifi network names are non-reversibly hashed out so that the actual wifi network names are hidden.	After you log into the Mood Study app for the first time
Network Sensor: Tracks the network your phone is connected to and the network type (data plan versus wifi network)	Network names are non-reversibly hashed out so that the actual names are hidden.	After you log into the Mood Study app for the first time

<u>Battery Charge:</u> Measures when the phone is charging and how full the battery is.	There is minimal risk that this information can be used to identify you	After you log into the Mood Study app for the first time
<u>Screen State</u>: Measures when your phone's screen is on or off.	There is minimal risk that this information can be used to identify you	After you log into the Mood Study app for the first time

Some information collected may be analyzed with Google tools. For example, information related to your location may be labeled with additional location data from Google about the types of businesses or places nearby. These analyses will be performed after completion of the study to see if they can help predict mood changes. These analyses will not be used to identify you personally, although they may inadvertently reveal information that could identify you, for example the types of places you have visited. Additional information regarding the privacy and security of your data can be found in the **"CONFIDENTIALITY"** section.

You may be contacted by the User Support Center if the Mood Study App on your phone is not collecting passive data properly.

STUDY DURATION
If you agree to participate in this study, your participation will last up to 12 weeks.

RISKS AND DISCOMFORTS

When you agree to create a Study Profile and use the Mood Study App, you provide Verily and the Mood Study Team with access to information about you. Verily Life Sciences will keep health information on secure Google and third party systems with many layers of protection. However, there is risk that someone could get unauthorized access to the information stored about you. Taking part in this study requires the use of one or more of an external study websites, mobile applications, messaging, and email. Because some of these systems are developed and managed externally, there is no guarantee that they are free of risk. Additional information regarding the privacy and security of your data can be found in the **"CONFIDENTIALITY"** section.

By using the Mood Study App, you may be exposed to the following additional risks:

- You may feel uncomfortable answering some of the assessment questions or you may feel fatigued by an assessment. You may decline to answer any question or discontinue the assessment at any time.
- Your responses to some assessments may be seen by others outside of the study, if someone other than yourself is able to access your phone (for example, if your phone is not locked via password protection and you leave it out of your care). You should not use someone else's device to participate in this study.

NEW INFORMATION
You will be told about any new information that becomes known to researchers that might change your decision to be in this study. You may be asked to sign a new consent form if this occurs.

BENEFITS
There is no direct benefit to you for participating in this study; however, the information obtained in this study may be helpful to others in the future.

COSTS
The study apps and assessments will be provided to you at no cost.

Depending on your mobile phone plan, you may incur charges such as receiving study text messages and data charges for accessing the Mood Study Web Portal and Mood Study App.

RESEARCH RESULTS
You will be able to view your PHQ-9 scoring results immediately after you complete the survey. This information is not meant to be used to make treatment decisions. Your PHQ-9 score will be displayed on the screen but not saved for later viewing. Once you exit the survey, you will no longer be able to see your PHQ-9 score.

You may also be able to view your responses to the assessments collected daily and every three days, as listed in the Procedure Section. You will not be able to change any of your responses, you will only be able to view responses which were previously submitted. You responses can be viewed through the Mood Study App for approximately 48 hours following completion of the assessment.

You will <u>not</u> have access to additional information collected nor will you receive feedback about your completed tasks, with the exception of PHQ-9 results which are meant to be informative only.

PAYMENT FOR PARTICIPATION
You will be compensated for your participation in this study. When you complete all required tasks for the week, you can expect weekly compensation in the range of $*[Min amount to be specified depending on compensation cohort]* to $*[Max amount to be specified depending on compensation cohort]* on any given week. Your compensation will vary from week to week, depending on what tasks you are asked to complete each week. You will be paid up to a maximum of $*[Amount to be specified depending on compensation cohort]*. If you do not complete the entire study, you will still be paid for the tasks you have completed.

Verily will work with a third party (which means they are independent from the study Sponsor) payment vendor to compensate you. You will be paid through a physical gift card that we will mail to you using the mailing address provided. This gift card may be used anywhere Visa is accepted. Your gift card will be remotely loaded with cash based on the study tasks you've completed. The Mood Study Team will aim to reload your gift card, at a minimum, every 4 weeks.

ALTERNATIVE TREATMENT
Taking part in this study is your choice. Since this study is not therapeutic in nature, the only alternative to participation is not to participate in this study.

CONFIDENTIALITY

Verily is helping to build and run some of the technology and systems used to develop your Study Profile and to conduct the Mood Study. Verily will keep your health information and data

secure and confidential on Google and third party systems with many layers of protection. Your personally identifiable data will be accessed only by study staff. Data used for purposes of conducting research and analysis will be used without identifiable reference to you. We may share research results or analyses with third parties, but will not share any details that can identify you personally. While Google provides the computing, analytics, and data handling power, Google will not sell your information for advertising.

Google's app store, called Play Store, will receive your email address and will log and track when you download the Mood Study App. This information will be stored in a secure and confidential manner, as described in the Play Store Terms of Use.

The payment vendor will receive information about you in order to compensate you for your participation in the study. This may include your name, email address, mailing address, and your date of birth. This information will be kept secure and confidential using data encryption methods standard to banking institutions.

Officials from the Mood Study team or its designees, qualified Institutional Review Boards (IRBs), or the state or federal government, such as the FDA, may review your information to make sure things are being done ethically and in a way that is consistent with this consent form.

The Mood Study team or its designees may be required to release information that identifies you to federal officials, if ordered to do so by a court or judge. The Mood Study team will make every effort to protect and maintain in confidentiality all information that may identify you, however if required by law or court order, your information may be released.

Once enrolled in the study, you may contact the Verily User Support Team for technical support. If you do so, you may need to provide your name, street address, email address and phone number to Verily so someone can assist you. This information will be used only to respond to your questions, if needed.

COMPENSATION FOR INJURY
The Mood Study App does not have any direct participant contact, therefore, a research related injury is not anticipated.

If you believe you are injured as a direct result of using the Mood Study App, you will be responsible for seeking medical treatment. Verily Life Sciences LLC will not provide medical treatment or compensate you for any costs incurred for medical treatment.

If you are injured as a result of this study, you do not give up your right to pursue a claim through the legal system.

VOLUNTARY PARTICIPATION AND WITHDRAWAL
Your participation in this study is entirely voluntary. You may decide not to participate or you may leave the study at any time. Your decision will not result in any penalty or loss of benefits to which you are entitled. If you decide to participate, you are free to withdraw your consent and refuse to participate at any time.

If you decide to withdraw from this study, you may do so by calling User Support at (844) 641-8390 between 8am and 11pm Eastern Time, Monday through Friday. When you withdraw from the study, you will delete the Mood Study App from your phone and no new health information identifying you will be gathered after that date. Information that has already been gathered, including information in your Study Profile, may still be used for research study purposes. If you withdraw your consent, your Study Profile will be archived in de-identified form and you will not be contacted for participation in future studies.

Your participation in this study may be stopped at any time by the study Investigator or the Sponsor without your consent for any reason, including:

- if you do not consent to continue in the study after being told of changes in the research that may affect you;
- if the study is cancelled;
- if you do not fulfill the study requirements; or
- any other reason believed to be in your best interest.

SOURCE OF FUNDING FOR THE STUDY
The Sponsor, Verily Life Sciences LLC, is paying to conduct this study.

FINANCIAL DISCLOSURE
Honor Hsin, MD PhD, the Principal Investigator, and the study staff are employees or consultants of the Sponsor. The Principal Investigator also owns equity in Verily Life Sciences LLC.

QUESTIONS
Contact the User Support Center at (844) 641-8390 between 8am and 11pm Eastern Time, Monday through Friday, for any of the following reasons:

- if you have any questions about your Study Profile,
- if you have any questions about your participation in this study,
- if you feel you have had a research-related injury, or
- if you have questions, concerns or complaints about the research.

The Principal Investigator can be reached by contacting the User Support Center at (844) 641-8390 between 8am and 11pm Eastern Time, Monday through Friday.

If you have questions about your rights as a research subject or if you have questions, concerns or complaints about the research, you may contact:

Western Institutional Review Board® (WIRB®)
1019 39th Avenue SE Suite 120
Puyallup, Washington 98374-2115
Telephone: 1-800-562-4789 or 360-252-2500
E-mail: Help@wirb.com.

WIRB is a group of people who independently review research.

WIRB will not be able to answer some study-specific questions, such as questions about appointment times. However, you may contact WIRB if the research staff cannot be reached or if you wish to talk to someone other than the research staff.

Do not sign this consent form unless you have had a chance to ask questions and have gotten satisfactory answers.

If you agree to be in this study, you will receive a signed and dated copy of this consent form for your records.

COMMERCIAL ISSUES

By participating in this study, you are agreeing that your data may be used for research or commercial purposes. If used commercially, you will not earn financial profit from commercial development. You will not have any rights to licensing from any products that may result from participating in this study.

In the course of your participation in this study, you may receive information (excluding any information relating to your health and your participation) about Verily's potential products and/or services that Verily considers confidential ("Verily Confidential Information"). You may use Verily Confidential Information only for this study. You must use reasonable care to protect Verily Confidential Information and to prevent any unauthorized use or disclosure of Verily Confidential Information. Verily Confidential Information does not include information that: (a) was known to you without restriction before this study; (b) is publicly available through no fault of yours; or (c) is independently developed by you. You may disclose Verily Confidential Information when compelled to do so by law if you provide reasonable prior notice to Verily, unless a court orders that Verily not be given notice.

CONSENT

I have read this consent form (or it has been read to me). All my questions about the study and my part in it have been answered. I freely consent to be in this research study.

By signing this consent form, I have not given up any of my legal rights.

____

Subject Name (printed)

____ __________________

Signature of Subject Date

<Participants will complete the below comprehension questions to ascertain their understanding of the study parameters. Participants must pass the quiz 100% to participate, but will be given an opportunity retake the quiz if they do not pass on the first attempt.>

1. I am a volunteer in this study and can exit at any time.

o True
o False

<A response of “true” returns the following:>
Correct!
Your participation is completely voluntary and you may withdraw from the study at any time.

<A response of “false” returns the following:>
Incorrect.
Your participation is completely voluntary and you may withdraw from the study at any time.

2. This study is 12 weeks long.

o True
o False

<A response of “true” returns the following:>
Correct!
The study runs for 12 weeks from the day you take your first assessment in the app.

<A response of “false” returns the following:>
Incorrect.
The study runs for 12 weeks from the day you take your first assessment in the app.

3. There is no clinician monitoring how I am doing in this study.

o True
o False

<A response of “true” returns the following:>
Correct!
There is no clinician monitoring your data during the study. Please contact your doctor if you need help or have any health concerns.

<A response of “false” returns the following:>
Incorrect.
There is no clinician monitoring your data during the study. Please contact your doctor if you need help or have any health concerns.

4. If I need help, I should contact my doctor.

<A response of “true” returns the following:>
Correct!
Please contact your doctor if you need help. There is no clinician monitoring your data during the study.

<A response of “false” returns the following:>
Incorrect.
Please contact your doctor if you need help. There is no clinician monitoring your data during the study.

5. The purpose of this study is to figure out the best way to collect information about depression from smartphones.

<A response of “true” returns the following:>
Correct!
The goal of this study is to determine if smartphones can help to collect information about depression.

<A response of “false” returns the following:>
Incorrect.
The goal of this study is to determine if smartphones can help to collect information about depression.

6. The mobile app you’ll use during this study collects the content of all the text messages you send (not receive) as well as other personal information such as your location and the names of other apps you use.

<A response of “true” returns the following:>
Correct!
The Mood Study App captures information including all the text messages you send, your location, and names of apps you use. A full description of the information collected can be found in your informed consent form, which you can access at any time by logging into this site.

<A response of “false” returns the following:>
Incorrect.
In this study, the Mood Study App collects the content of all the text messages you send, your location, summary data on the apps you use, and other sensitive information on your phone. A full description of the information collected can be found in your informed consent form, which you can access at any time by logging into this site. You can also take another look at the informed consent before you sign it and enroll in the study.
