# Supplementary material for: Toward a Mobile Platform for Real-world Digital Measurement of Depression: User-Centered Design, Data Quality, and Behavioral and Clinical Modeling
Source: JMIR Ment Health. 2021 Aug 10;8(8):e27589. doi: 10.2196/27589 (PMC8386379; doi:10.2196/27589)
Supplement: Multimedia Appendix 3 [file mental_v8i8e27589_app3.pdf]

User experience research and user-centered design

This document provides, in chronological order, an overview of the User Experience Research activities and studies conducted and describes how they guided the development of the study app.

Initial problem definition interviews with external mental health professionals

We conducted interviews and focus groups with 8 mental health professionals from a local psychiatric hospital to discover user needs around understanding and tracking depression using a digital platform.

Design feedback interviews

We collected feedback from mental health professionals on specific design ideas through interviews to understand likely reactions from a depressed population to things like: specific GIF images that may be triggering or inappropriate, tonal acceptability, and sensitivity to different payment mechanisms (e.g., micropayments per task vs. lump sum payment).

A/B testing of design and engagement with target users

We conducted 4 remote, A/B-style, within-participant comparison studies with volunteers on Amazon's Mechanical Turk platform. Participants were unaware of the identity of the issuer of the tasks, and we did not collect any personally identifiable information from the participants. The goal of these experiments was to assess user reactions to different iterations of the study app's irreverent tone choice, inclusion of GIFs and emojis, and other design choices that would not be typical for a depression-related study app. At the time the results were interpreted numerically and directionally (i.e., no statistical analysis was conducted) and we will therefore present the results in the same way here. With each iteration of the design, we aimed to maximize potential engagement yet minimize perception of unprofessionalism.

Study 1: Fox vs. Robot

In the first study, we recruited 50 participants with depression (self-reported on Mechanical Turk) to compare two versions of the app. FOX was a more casual, animated version; ROBOT was more formal in tone and presentation (see example of user interface below). Each participant tried out both versions in a randomized order, then answered questions on engagement, appropriateness, and overall preference.

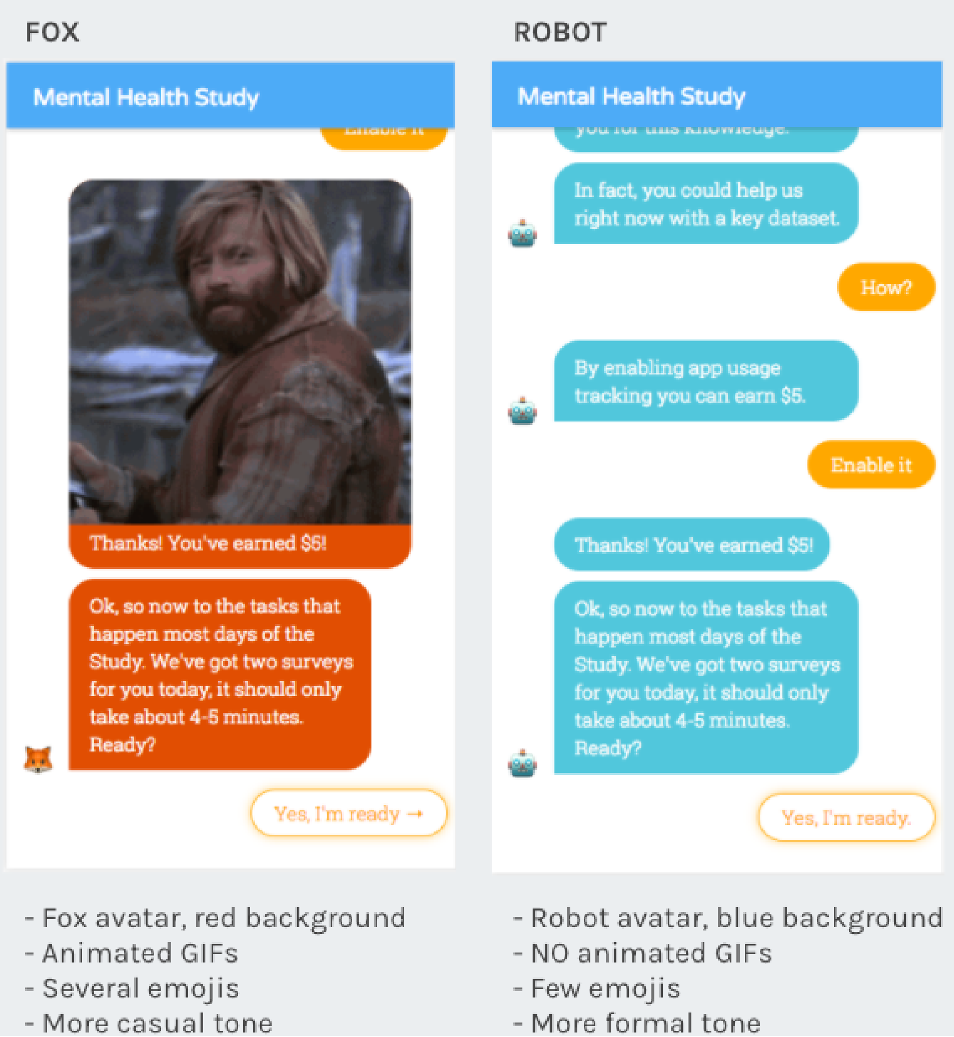

When asked which words best describe this version from a list of 10 words, participants answered as follows.

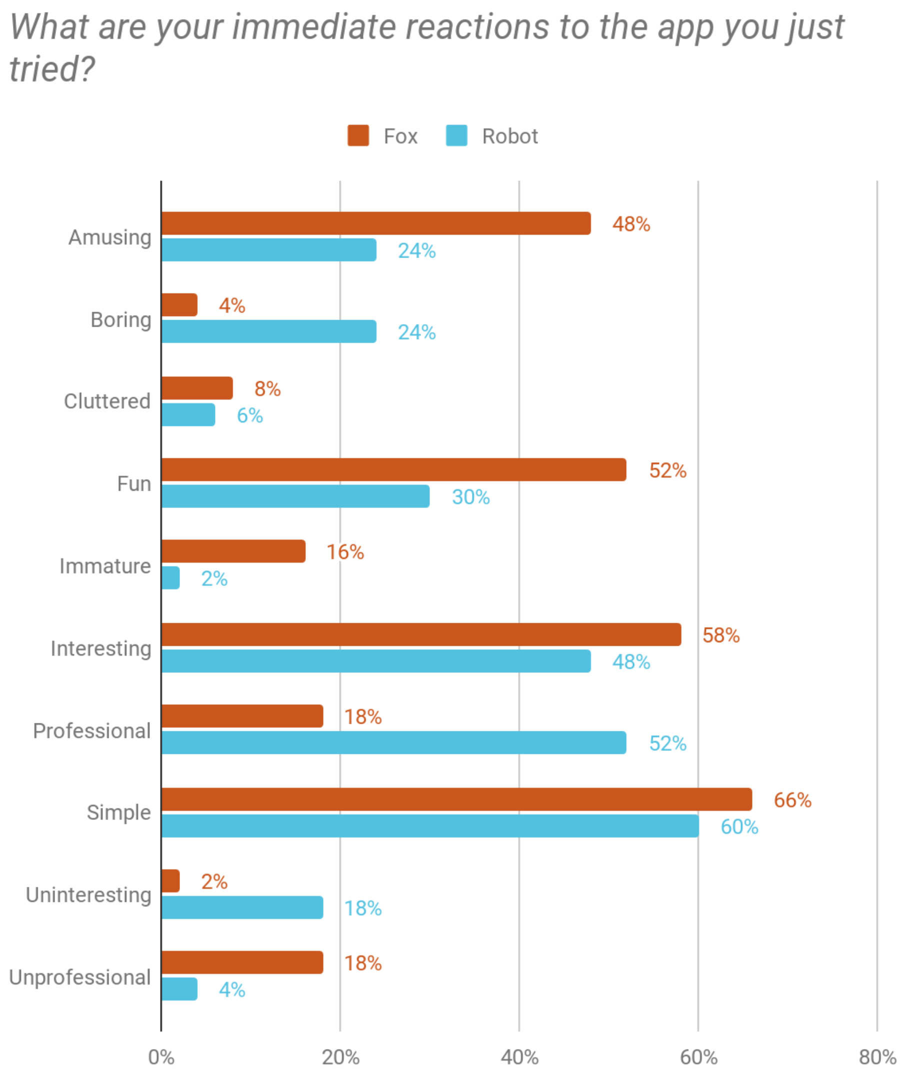

A larger percentage of participants described FOX as fun (52%) compared to ROBOT (30%), while ROBOT was considered more professional (52%) than FOX (18%). FOX received more endorsements of immature (16%) and unprofessional (18%) than ROBOT (2%, 4%), perhaps due to the more playful tone and imagery of FOX.

When asked how engaging or boring the different versions were, FOX was rated as more engaging (90% extremely, moderately, or slightly engaging) than ROBOT (78%).

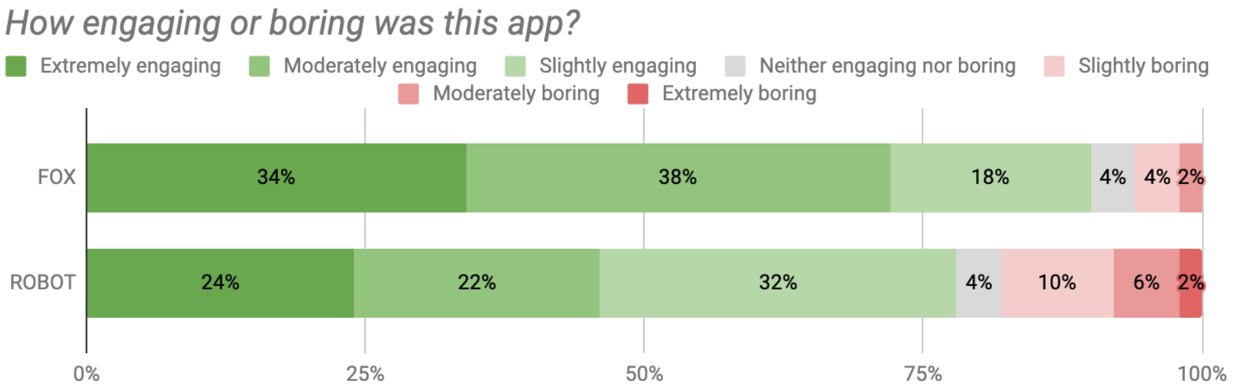

ROBOT's tone was considered completely acceptable by more participants (68% vs. 46% for FOX), but the sum of all acceptable ratings was the same (84%) for both FOX and ROBOT.

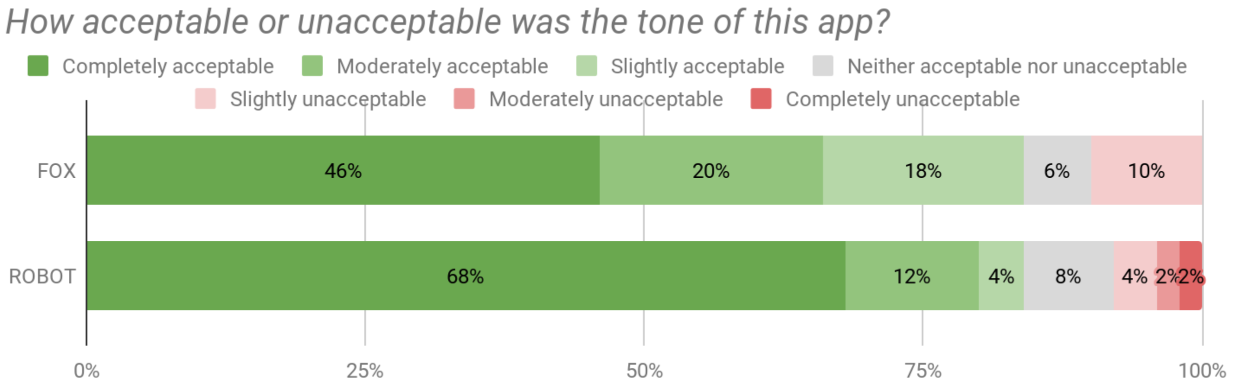

Asking about a preference for one of the versions, FOX was preferred by 66% of participants (the sum of greatly, moderately, and somewhat preferred), ROBOT by 26%.

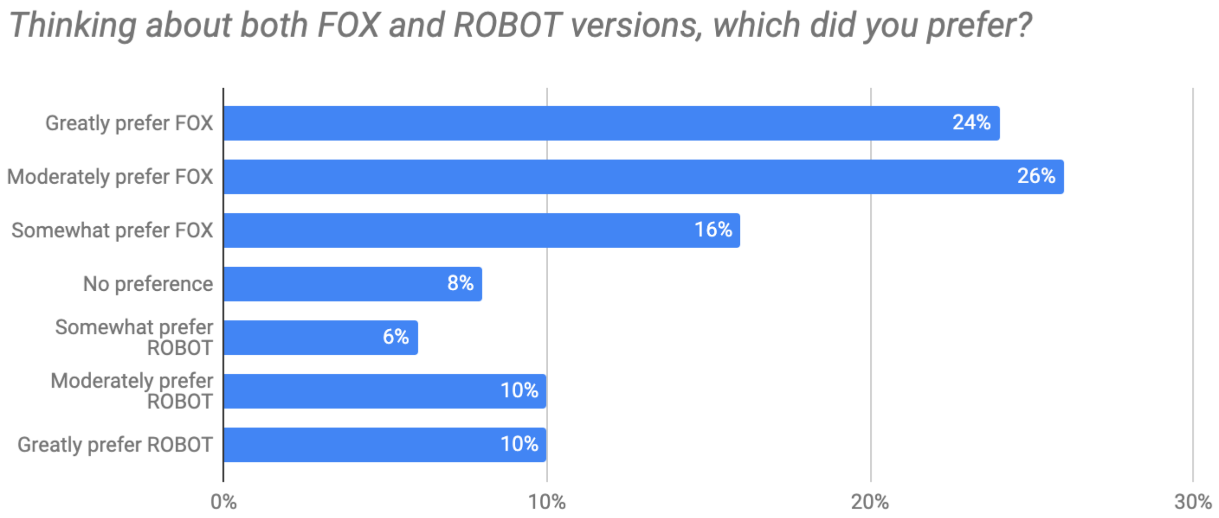

When asked about the GIFs (only present in FOX), 70% reported liking the pictures. However, 16% disliked the GIFs.

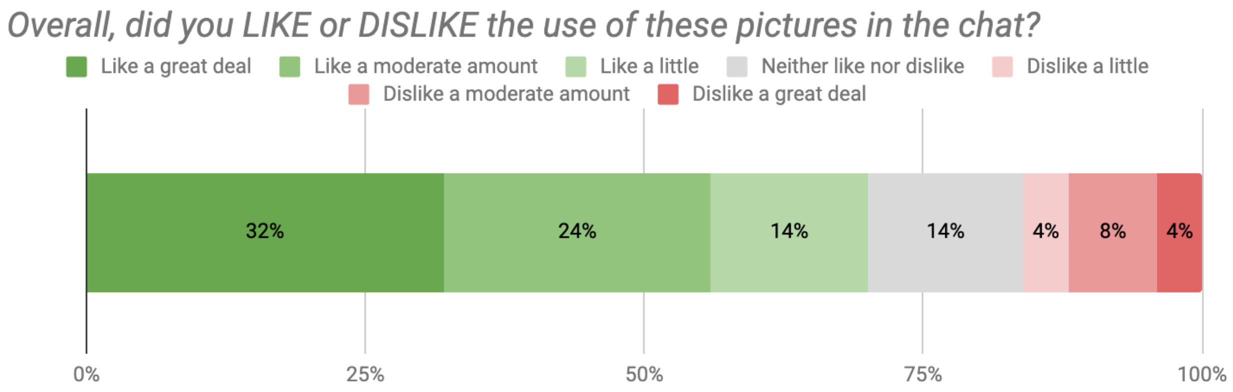

Conclusions from this study included that users prefer the FOX version, perhaps due to its friendlier avatar and inclusion of more casual GIFs. Participants rated the tone of both versions similarly, however, more participants found ROBOT to be completely acceptable in tone. Regarding the avatars, in open-ended feedback (not shown in this report) users spoke more positively about FOX and far more negatively about ROBOT. Thus, for the next study, we planned to replace the avatars with more similar icons to determine how this impacts users' perceptions of the GIFs and lighter tone.

Study 2: Red vs. Blue

In the second study, we recruited another 50 participants with self-reported depression on Mechanical Turk to compare two new versions. These versions replaced the fox and robot avatars with a small flame icon (red or blue) to focus user evaluation on content and tone, rather than preference for the avatar. All other aspects of the two apps were identical to Study 1, meaning that RED used a more playful tone and included GIFs, whereas BLUE was more formal in tone and used no GIFs (see example below).

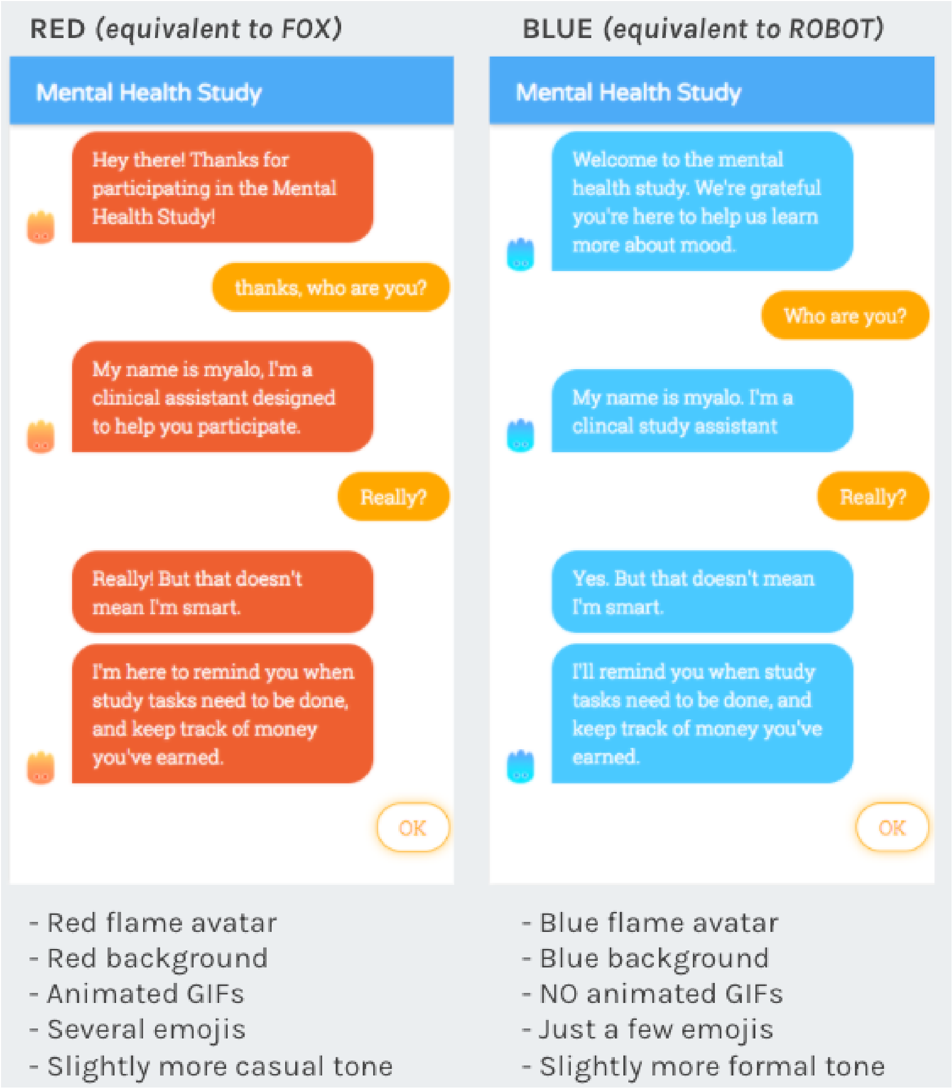

Participants were again asked to pick from a list of words which words best describe this version, with the following result.

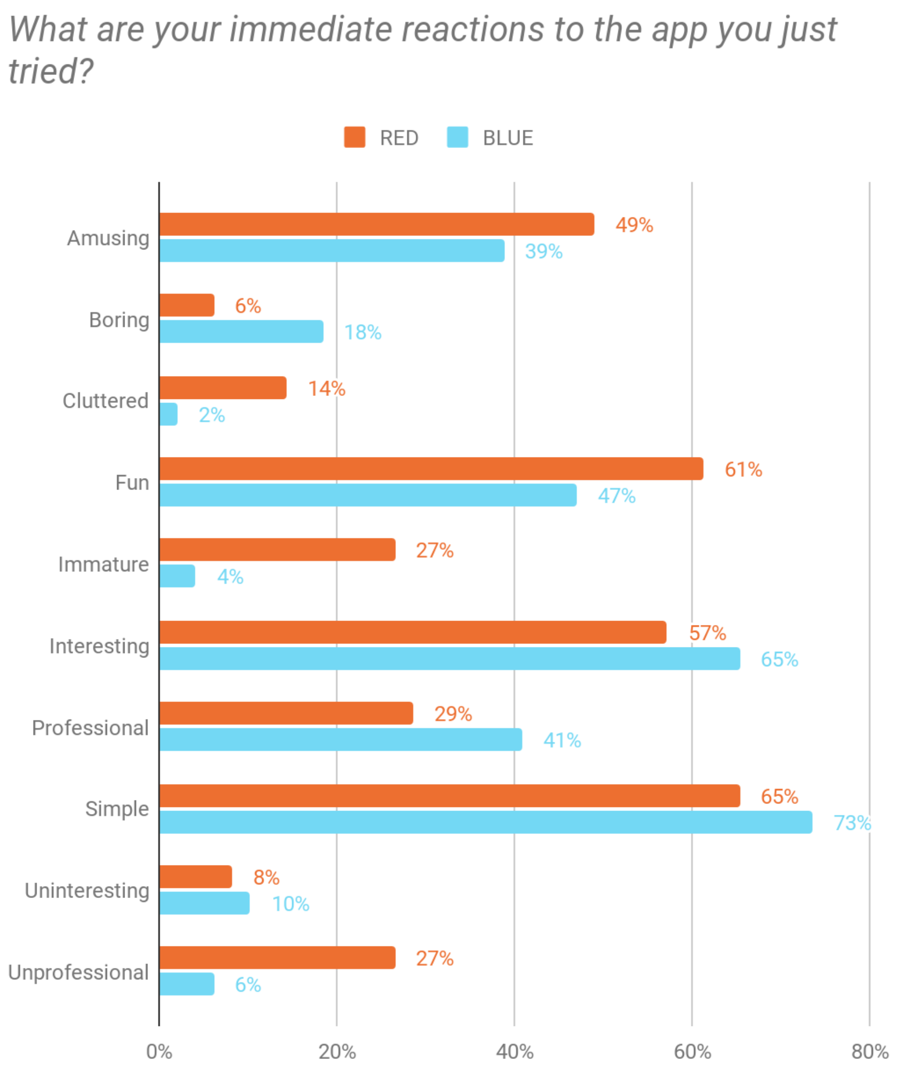

As before, a subset of participants did not like the more playful tone and imagery of RED (similar to FOX), which yielded more endorsements of immature (27%) and unprofessional (27%) than BLUE (similar to ROBOT) (4%, 6%).

When asked how engaging or boring this app was, participants rated both versions similarly: 76% categorized RED as extremely, moderately or slightly engaging compared to 73% for BLUE. However, RED received more endorsements of extremely engaging (35% vs. 24%).

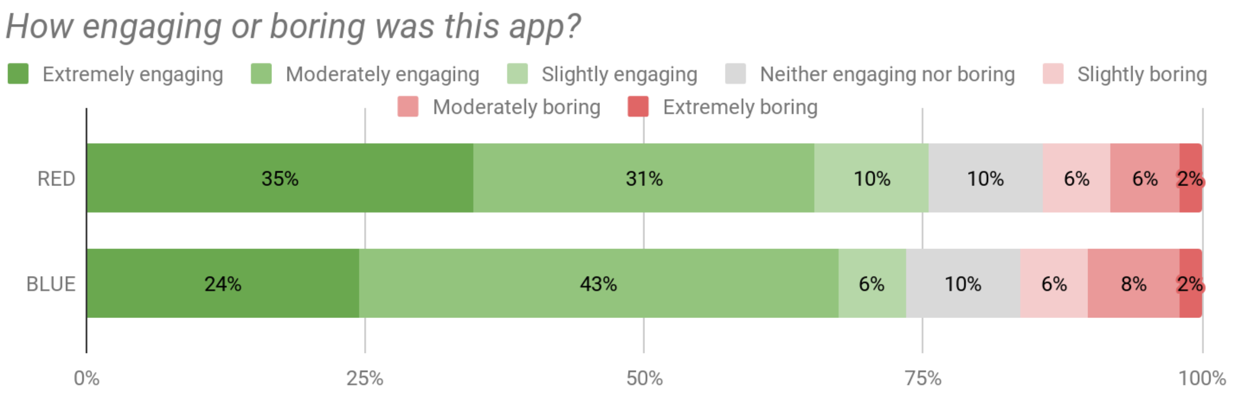

More participants endorsed BLUE as completely acceptable in tone (59% vs. 49% for RED). RED's tone was rated as unacceptable by a higher percentage (14%) compared to BLUE (4%), and open-ended comments highlighted the GIFs as driving this opinion.

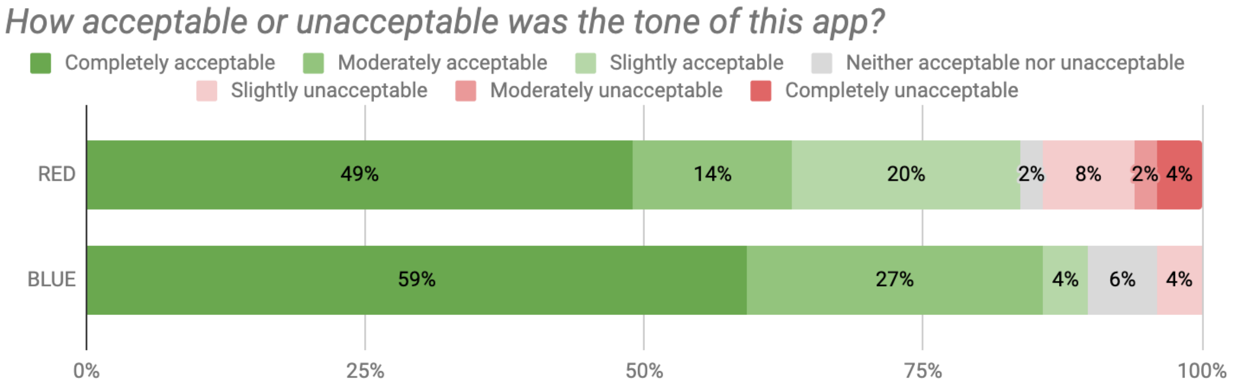

Still, RED was slightly preferred (51% greatly, moderately, or somewhat preferred RED) over BLUE (39%), although BLUE had the largest percentage of greatly prefer endorsements.

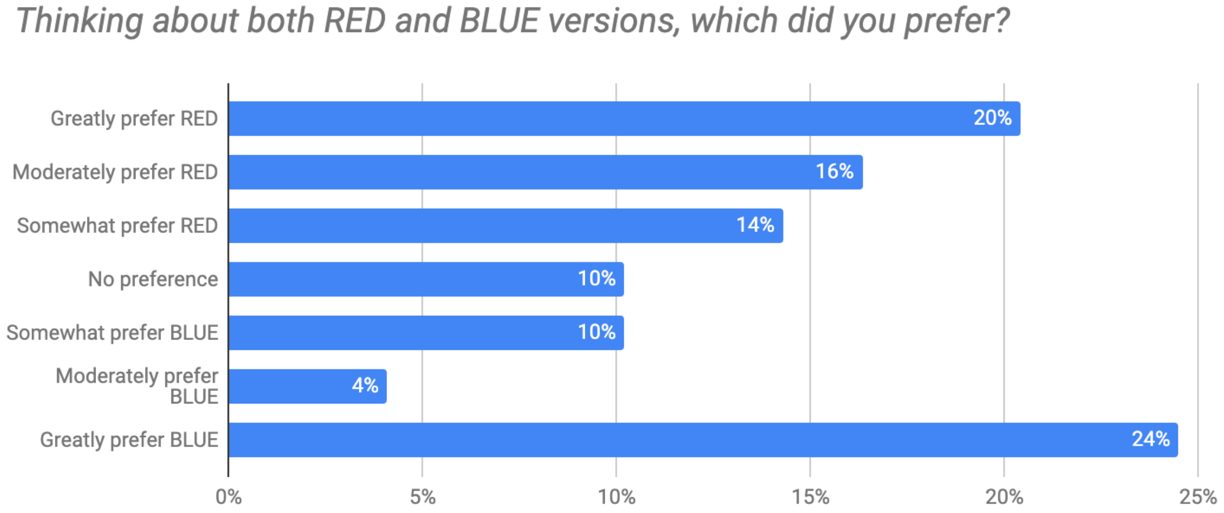

Overall, with respect to GIFs, we continued to see a partial preference for the version with animated GIFs, but slightly weaker than for Study 1 (66% preferred FOX over ROBOT, 51%

preferred RED over BLUE, though note that these answers came from different samples).Yet, those who prefer the non-GIF version feel strongly opposed to GIFs, finding the memes inappropriate in this context as reported in the open-ended comments.

However, in these two experiments the users were simulating the experience of multiple days of chat interactions during the course of a few minutes, and seeing 3 GIFs during this time may have created the feeling of an overly GIF-heavy experience. Since frequency in the actual study would be closer to 1 GIF per week, we planned for the next study to reduce the GIF exposure to a single instance.

Regarding the avatar, in this study there were far fewer mentions of the avatars (more abstract flame icons) in the open-ended questions, suggesting that users may be ascribing more personality to an animal or more defined character. Users may be more forgiving of a personality rather than a more faceless icon. Also, in this study using the flame, tasks may be seen to be coming from a company or study, rather than from a persona.

Study 3: X vs. Y

In the third study, we recruited another 50 participants to test a toned-down version of the GIF app with only one GIF (X) versus a version with no GIFs (Y). Both versions used the same avatar and background color (blue) so that these variables could not influence participants' perceptions.

We wished to see if a single GIF might retain overall preference for that version while mitigating concerns of unprofessionalism from users preferring a more formal approach.

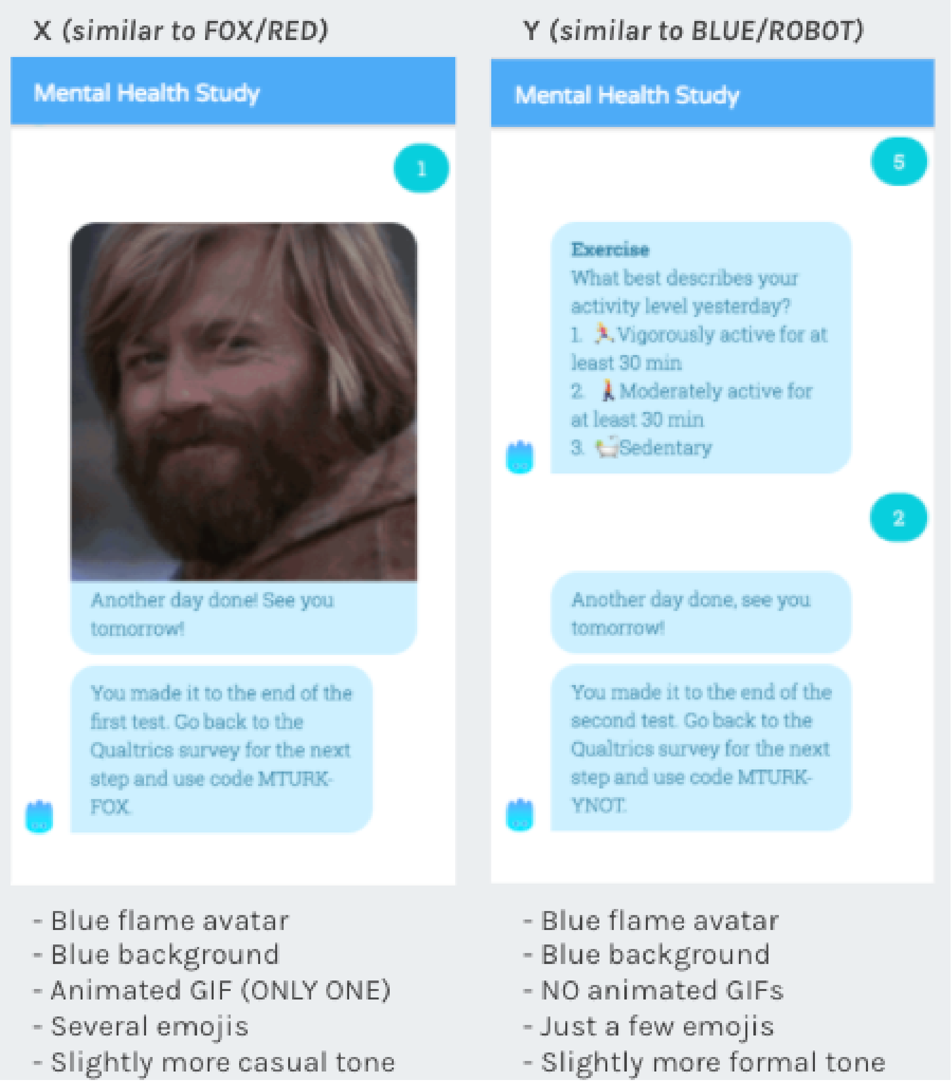

When asked which words best describe this version, participants reported the following:

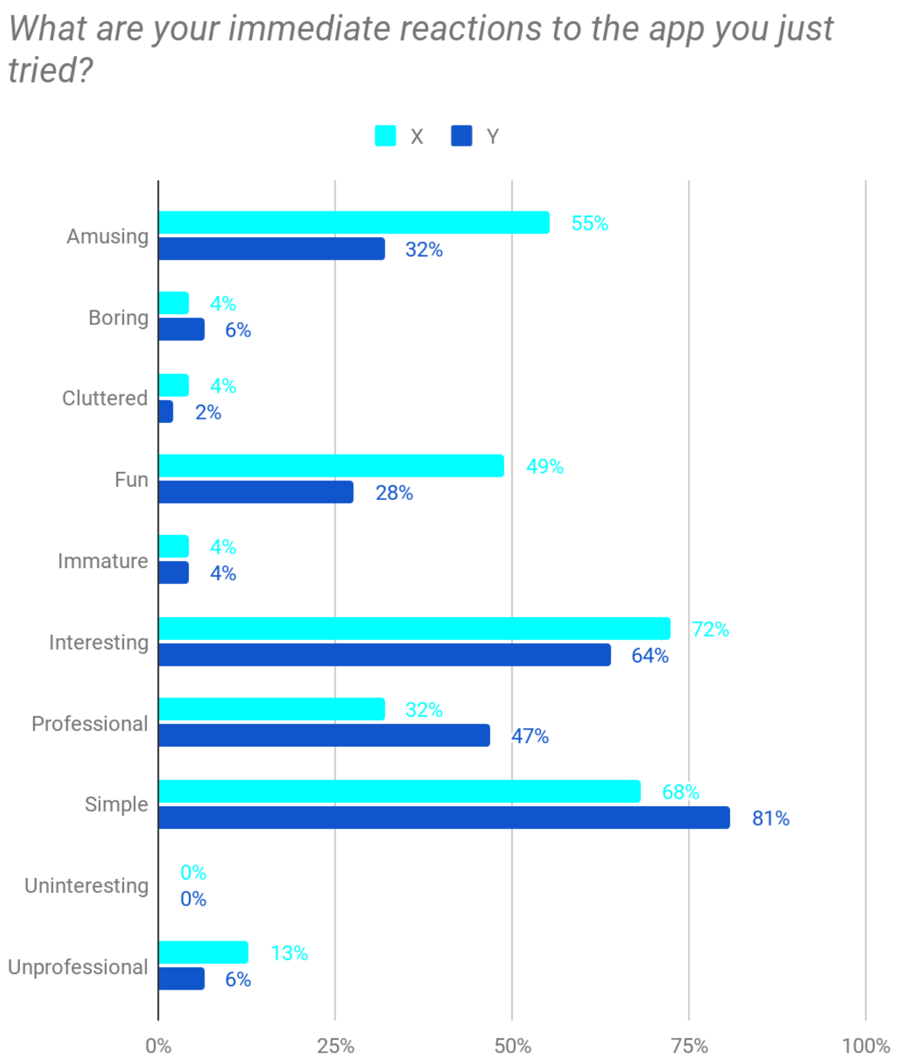

The reduced-GIF version (X) dropped the reports of immature (4%) and unprofessional (13%), as compared to the RED version from Study 2 which contained 3 GIFs (27% immature, 27% unprofessional, though note that those ratings came from different participants). X also showed greater endorsements of amusing (55%) and fun (49%) than the no-GIF version (Y, 32% amusing, 28% fun). It thus appeared that reducing the frequency of GIFs mitigated concerns of unprofessionalism.

Versions X and Y were rated similarly high in terms of engagement (91% vs. 87%), though version X with one GIF was rated as extremely engaging by more users (23% vs. 15%). This suggests that even an infrequent inclusion of a GIF may capture attention in an engaging way.

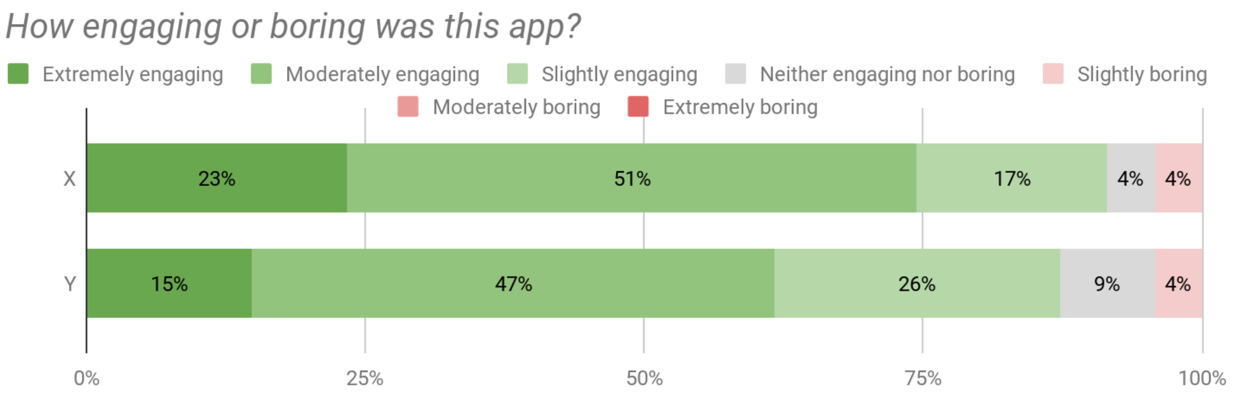

Both versions were rated as highly acceptable (X: 89%, Y: 87%), though the non-GIF version (Y) received slightly more completely acceptable ratings (49% vs. 43%).

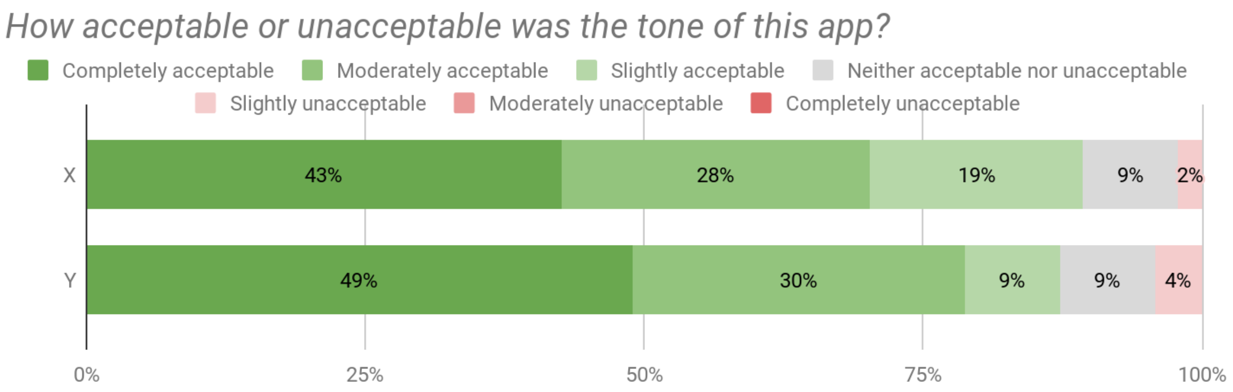

Importantly, version X with one GIF was preferred (64%) over version Y without any GIFs (18%).

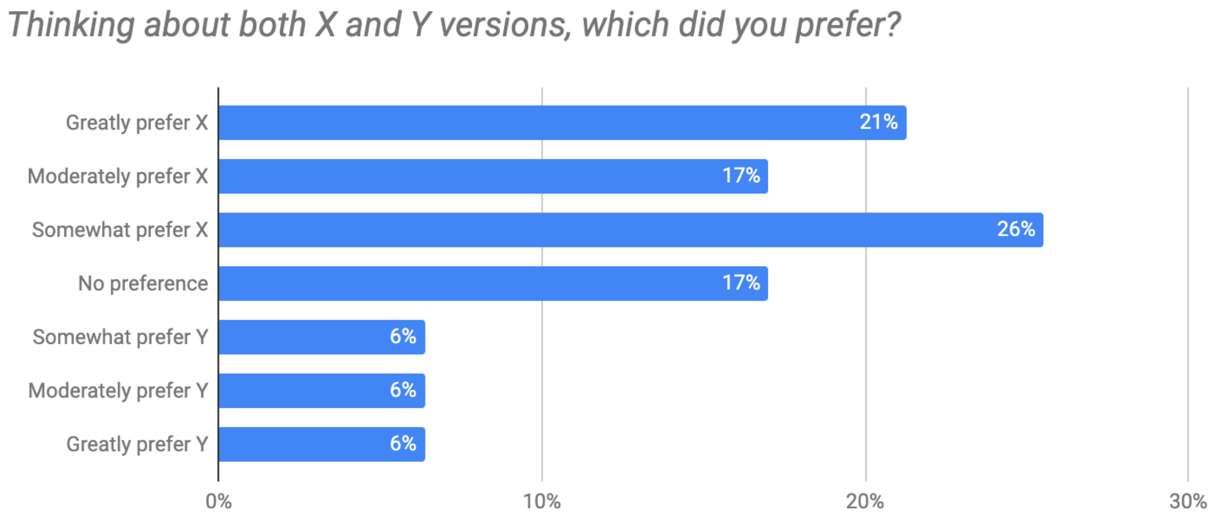

Still, 17% disliked the single GIF in X a little or to a moderate amount.

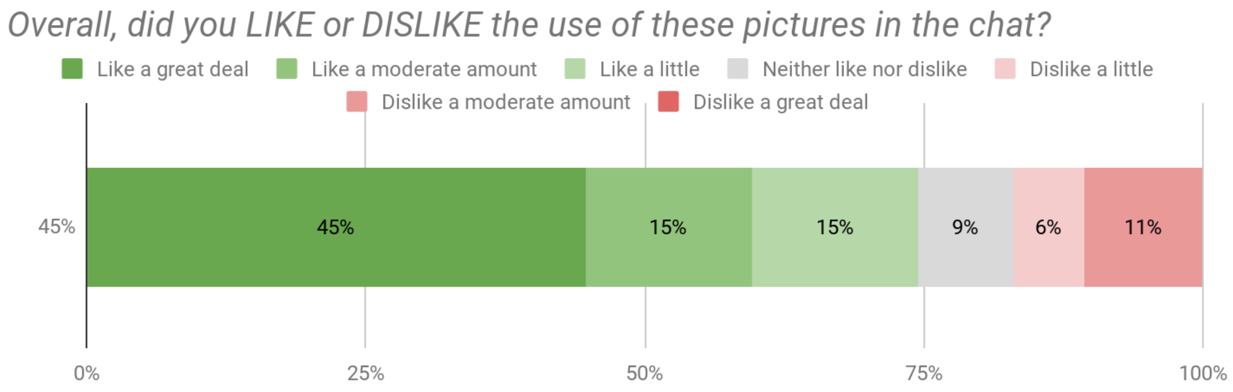

Study 4: Fox vs. Owl vs. Dog vs. Flame

We recruited 100 participants to try one of four prototypes with new avatar illustrations: owl, fox, flame, and dog. All other app characteristics were identical between the versions. Afterwards, participants were asked to pick among all 4 for their preference.

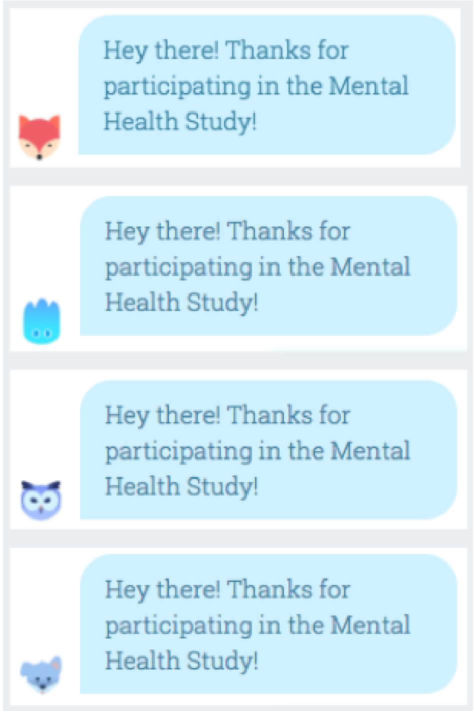

Participants described the four avatars with these words that could be picked from a list:

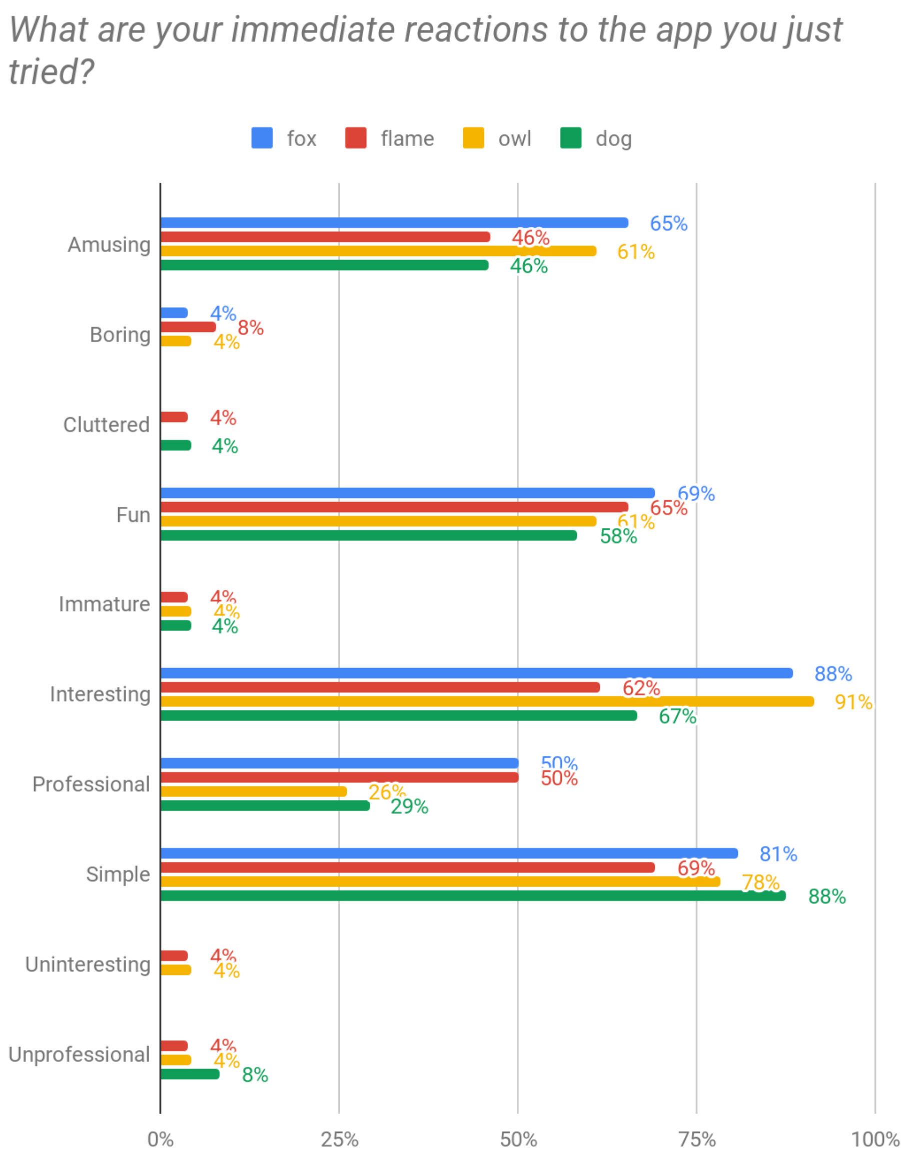

Fox and owl were rated more amusing and interesting than flame and dog, and none of them yielded high endorsements of immature or unprofessional.

Participants reported all avatars to be similarly engaging, and their tone equally acceptable (data not shown). Note that the language used and the number of GIFs was the same for all four avatars.

Lastly, we directly asked users which avatar they preferred. The owl avatar won out by a small margin.

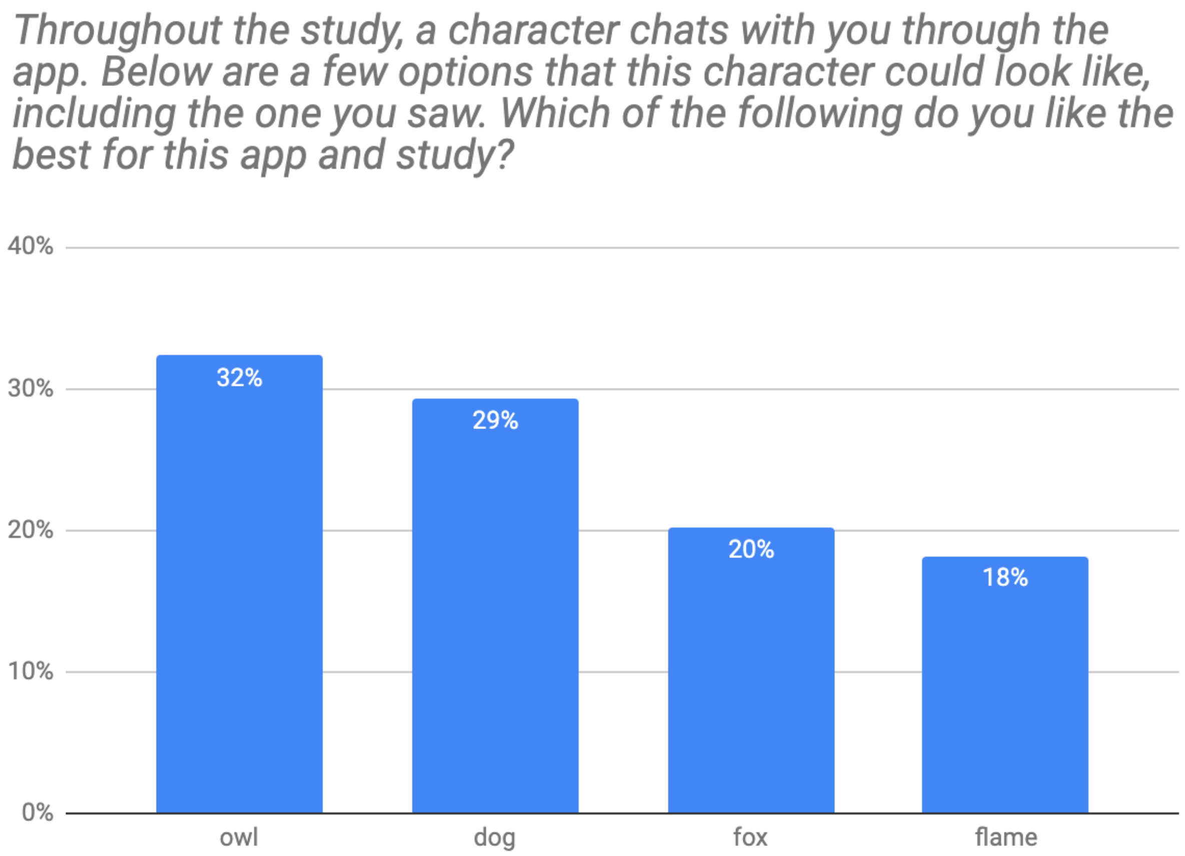

After conducting these four experiments we landed on an app design with a more casual tone, a blue background, about 1 GIF per week, and an owl as the avatar. For the final app design see Figure 1 in the main text.
