# Supplementary material for: Toward a Mobile Platform for Real-world Digital Measurement of Depression: User-Centered Design, Data Quality, and Behavioral and Clinical Modeling
Source: JMIR Ment Health. 2021 Aug 10;8(8):e27589. doi: 10.2196/27589 (PMC8386379; doi:10.2196/27589)
Supplement: Multimedia Appendix 4 [file mental_v8i8e27589_app4.pdf]

Study population characteristics

Table S1 gives additional demographic summaries of the enrollment cohort and minimally sufficient data cohort. Note that depression status is not available for all participants from the enrollment survey cohort because if participants answered earlier parts of the enrollment survey in a way that made them ineligible for the study, they did not move on to answer the PHQ-9 questionnaire.

Table S1: Detailed demographics of a) all participants that completed the enrollment survey (n=2,360) and b) all participants that were found eligible, enrolled, and provided a minimum of data (Figure 2), shown here separately for depressed and not depressed participants (based on baseline PHQ-9 survey at enrollment).

		**Completed enrollment survey (n=2360)**		**Participants with minimally sufficient data (n=384)**			
				**Depressed (n=313)**		**Not Depressed (n=71)**	
		**Count**	**Percent**	**Count**	**Percent**	**Count**	**Percent**
Cohort	Depressed (PHQ-9 >=10)	506	53.5%	313	100%	0	0%
	Not Depressed (PHQ-9 < 10)	440	46.5%	0	0%	71	100%
Age	18-29	855	36.2%	123	39.3%	26	36.6%
	30-39	674	28.6%	90	28.8%	22	31%
	40-49	429	18.2%	56	17.9%	14	19.7%
	50-59	284	12%	36	11.5%	8	11.3%
	60-69	93	3.9%	7	2.2%	0	0%
	70-79	20	0.8%	1	0.3%	1	1.4%
	Other	5	0.2%	0	0%	0	0%
Education	High School	638	27%	93	29.7%	9	12.7%
	Some College	987	41.8%	128	40.9%	29	40.8%
	College	577	24.4%	77	24.6%	23	32.4%
	Graduate School	158	6.7%	15	4.8%	10	14.1%
Employment Status	Full-time	738	31.3%	74	23.6%	32	45.1%
	None	938	39.7%	151	48.2%	16	22.5%
	Part-time	408	17.3%	45	14.4%	14	19.7%
	Student	224	9.5%	33	10.5%	7	9.9%

	Volunteer	52	2.2%	10	3.2%	2	2.8%
Income	less than $10,000	1319	55.9%	198	63.3%	26	36.6%
	$20,000 - $39,999	627	26.6%	73	23.3%	18	25.4%
	$40,000 - $59,999	243	10.3%	24	7.7%	9	12.7%
	$60,000 - $79,999	92	3.9%	9	2.9%	8	11.3%
	$80,000 - $99,999	31	1.3%	3	1%	5	7%
	More than $100,000	48	2%	6	1.9%	5	7%
Relationship Status	Divorced	344	14.6%	54	17.3%	4	5.6%
	Married	660	28%	80	25.6%	28	39.4%
	Separated	87	3.7%	12	3.8%	2	2.8%
	Single	1205	51.1%	158	50.5%	37	52.1%
	Widowed	64	2.7%	9	2.9%	0	0%
Race	American Indian or Alaska Native	40	1.7%	12	3.8%	1	1.4%
	Asian	37	1.6%	5	1.6%	4	5.6%
	Black or African American	272	11.5%	29	9.3%	12	16.9%
	Native Hawaiian or Other Pacific Islander	6	0.3%	2	0.6%	1	1.4%
	Other	94	4%	14	4.5%	1	1.4%
	White	1911	81%	251	80.2%	52	73.2%
Sex At Birth	Female	2077	88%	285	91.1%	51	71.8%
	Male	283	12%	28	8.9%	20	28.2%
