# Supplementary material for: Toward a Mobile Platform for Real-world Digital Measurement of Depression: User-Centered Design, Data Quality, and Behavioral and Clinical Modeling
Source: JMIR Ment Health. 2021 Aug 10;8(8):e27589. doi: 10.2196/27589 (PMC8386379; doi:10.2196/27589)
Supplement: Multimedia Appendix 5 [file mental_v8i8e27589_app5.pdf]

Phone Types

Figure S1 contains a high level overview of phone types (manufacturer only) that were present in this study.

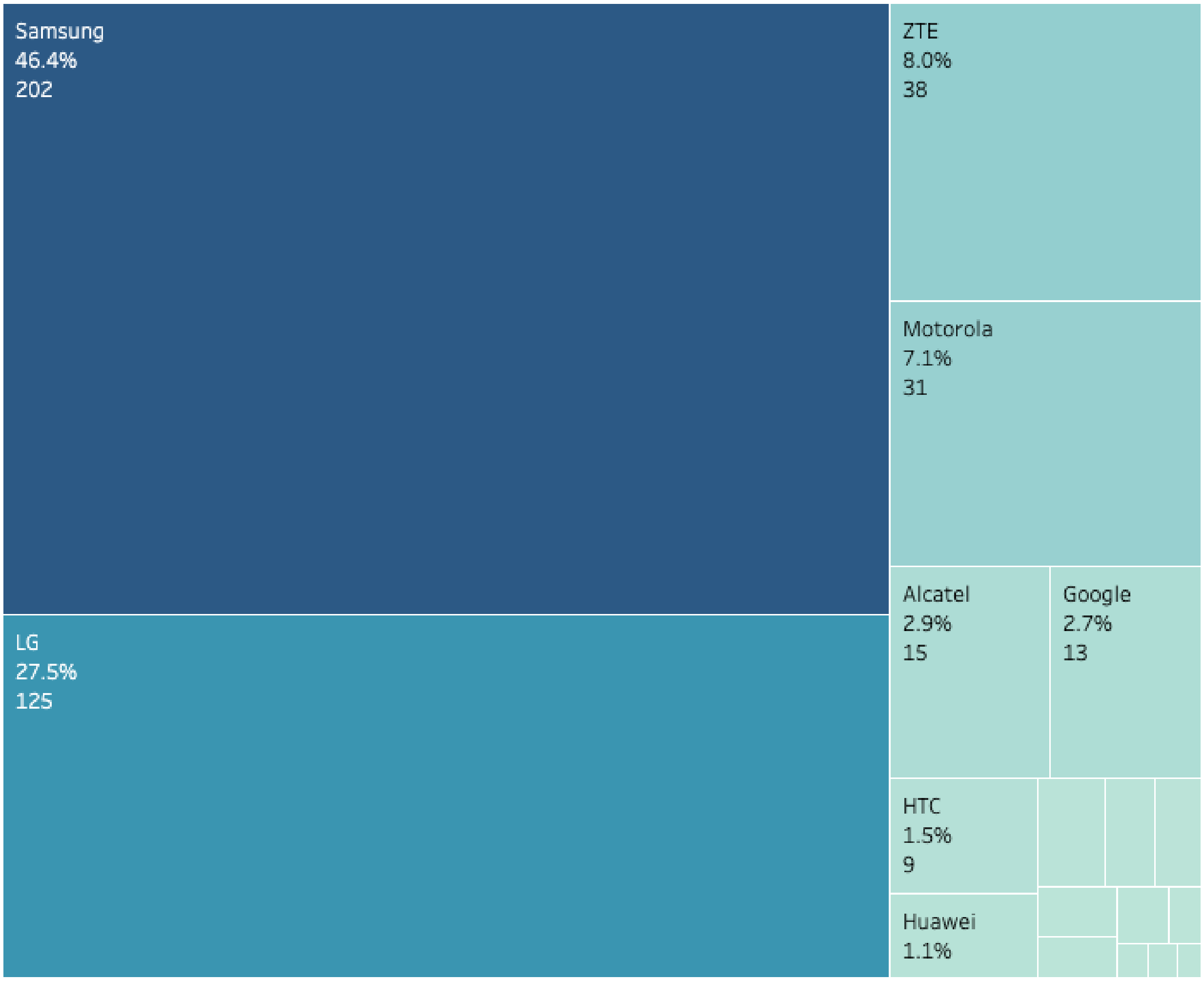

Figure S1: Phone type distribution where size of the rectangle corresponds to the percentage of days of all study days that this phone type was used. The integer below is the count of unique participants that ever used this phone type.

Table S1 contains a more granular breakdown of the phone types that were present in the study. Note that phone type can be null when we had no information about the phone type on a given day because the information from the Android device sensor was missing.

Table S1: Detailed breakdown of manufacturer, make, and model of all phone types present in the study.

Phone Type	% of all study days during which this phone type was used	Total number of days this phone type was used	Unique participants that used this phone type
Samsung Galaxy S7	7.4%	33,558	34
Samsung Galaxy S8	4.9%	22,120	20
Samsung Galaxy S7 Edge	4.0%	18,004	17
Samsung Galaxy S6	3.5%	15,694	16
Samsung Galaxy S8+	3.2%	14,574	14
LG Aristo	2.8%	12,502	15
LG K20 Plus	2.8%	12,600	13
Samsung Galaxy Note 8	2.5%	11,452	11
Samsung Galaxy S5	2.3%	10,556	10
LG Stylo 2 Plus	2.2%	10,080	10
LG Stylo 3	1.9%	8,568	9
Samsung Galaxy J3 Emerge	1.8%	7,938	8
ZTE Z Max Pro	1.8%	8,064	9
Motorola Moto E 4	1.7%	7,784	8
Samsung Galaxy J3	1.6%	7,084	7
ZTE Majesty Pro	1.4%	6,314	6
Samsung Galaxy J3 Prime	1.3%	5,740	6
LG Fortune	1.1%	4,802	5
LG Tribute HD	1.1%	5,096	5
Samsung Galaxy On5	1.1%	5,166	6
Google Pixel	1.0%	4,606	4
LG K7	1.0%	4,662	8
LG Nexus 5X	1.0%	4,620	6
LG Stylo 2	1.0%	4,466	4
Motorola Droid Turbo 2	1.0%	4,340	5
Samsung Galaxy Grand Prime	1.0%	4,662	7
Samsung Galaxy Note 5	1.0%	4,690	5
Samsung Galaxy S8 Active	1.0%	4,480	4
LG G5	0.9%	3,864	5
LG Stylo 3 Plus	0.9%	4,172	5
Motorola Moto G Play	0.9%	3,962	4

Samsung Galaxy Note 4	0.9%	3,976	5
LG Premier	0.8%	3,514	3
LG X Charge	0.8%	3,584	4
Motorola Moto G 4	0.8%	3,514	3
Motorola Moto Z Force Droid	0.8%	3,458	3
Samsung Galaxy J3 Luna Pro	0.8%	3,794	4
Samsung Galaxy S7 Active	0.8%	3,584	4
Google Pixel XL	0.7%	3,038	4
ZTE Blade Z MAX	0.7%	3,262	3
Google Pixel 2 XL	0.6%	2,492	3
LG Escape 3	0.6%	2,814	3
LG G6	0.6%	2,674	3
Samsung Galaxy S4	0.6%	2,814	3
Samsung Galaxy S9	0.6%	2,548	8
ZTE Maven 3	0.6%	2,618	3
Alcatel Pixi Avion	0.5%	2,338	2
Alcatel Pixi Unite	0.5%	2,380	2
Google Pixel 2	0.5%	2,282	2
HTC One M9	0.5%	2,352	2
LG G4	0.5%	2,338	2
LG Stylo 2 V	0.5%	2,338	2
LG Transpyre	0.5%	2,366	2
LG V10	0.5%	2,338	2
LG X Power	0.5%	2,198	3
Motorola Moto Z 2	0.5%	2,170	2
Samsung Galaxy Core Prime	0.5%	2,128	2
Samsung Galaxy J3 Eclipse	0.5%	2,352	2
Samsung Galaxy J7	0.5%	2,352	2
Samsung Galaxy J7 Sky Pro	0.5%	2,436	3
Samsung Galaxy Note 3	0.5%	2,352	2
Samsung Galaxy S6 Active	0.5%	2,352	2
Samsung Galaxy S6 Edge	0.5%	2,352	2
Samsung Galaxy S9+	0.5%	2,086	4
HTC 10	0.4%	1,610	2
LG Aristo 2	0.4%	1,624	3

LG G Stylo	0.4%	1,596	2
LG Rebel	0.4%	1,974	2
LG Tribute 2	0.4%	1,666	2
LG Tribute 5	0.4%	1,806	2
LG V20	0.4%	1,988	2
Samsung Galaxy Amp 2	0.4%	1,932	3
NULL (information about phone type was missing)	0.3%	1,246	60
Alcatel A30 Fierce	0.3%	1,582	2
Alcatel Flint	0.3%	1,176	1
Alcatel idealXCITE	0.3%	1,176	1
Alcatel Idol 3	0.3%	1,176	1
Coolpad REVVL Plus	0.3%	1,358	2
HTC Desire 626s	0.3%	1,148	1
Huawei Honor 6x	0.3%	1,176	1
Huawei Mate 8	0.3%	1,176	1
Huawei Nexus 6P	0.3%	1,134	1
Kyocera Hydro Wave	0.3%	1,176	1
LG Fiesta	0.3%	1,190	2
LG K10	0.3%	1,176	1
LG K20	0.3%	1,260	2
LG Optimus Zone 3	0.3%	1,568	2
LG Rebel 2	0.3%	1,176	1
LG Treasure	0.3%	1,358	2
Motorola Moto E 2	0.3%	1,176	1
Motorola Moto G 5S Plus	0.3%	1,176	1
Motorola Moto Z 2 Play	0.3%	1,176	1
OnePlus 5T	0.3%	1,176	1
Samsung Galaxy Express 3	0.3%	1,176	1
Samsung Galaxy J7 Perx	0.3%	1,274	2
Samsung Galaxy J7 Prime	0.3%	1,302	2
Samsung Galaxy Luna	0.3%	1,470	2
Sony Xperia M2	0.3%	1,176	1
Unimax U673c	0.3%	1,568	2
ZTE Citrine	0.3%	1,176	1

ZTE Jasper	0.3%	1,176	1
ZTE Majesty Pro Plus	0.3%	1,344	2
ZTE Max XL	0.3%	1,344	3
ZTE N818s	0.3%	1,162	1
ZTE Overture 3	0.3%	1,246	2
ZTE Prestige 2	0.3%	1,176	1
ZTE Z Five 2	0.3%	1,372	2
Alcatel Fierce 4	0.2%	826	1
Alcatel Idol 4	0.2%	1,078	2
Alcatel Pixi Theatre	0.2%	700	1
Essential PH-1	0.2%	728	1
HTC Bolt	0.2%	896	1
Kyocera Brigadier	0.2%	700	1
LG Fiesta 2	0.2%	1,064	1
LG Harmony	0.2%	896	1
LG K8 V	0.2%	994	1
LG K20 V	0.2%	1,092	2
LG Phoenix 2	0.2%	980	1
Motorola Droid Maxx 2	0.2%	1,120	1
Motorola Moto G 5 Plus	0.2%	1,036	2
Motorola Nexus 6	0.2%	980	1
OnePlus 1	0.2%	994	1
Samsung Galaxy J3 Mission	0.2%	826	1
Samsung Galaxy S5 Active	0.2%	826	1
ZTE Blade X Max	0.2%	1,120	1
ZTE Grand X 3	0.2%	1,120	1
ZTE Grand X 4	0.2%	1,064	1
ZTE Tempo X	0.2%	714	1
ZTE Z Five G	0.2%	700	1
Alcatel Dawn	0.1%	448	1
ANS UL40	0.1%	378	1
ASUS Zenfone 3 Laser	0.1%	420	1
Coolpad Catalyst	0.1%	406	1
Coolpad Defiant	0.1%	364	1
HTC Desire 530	0.1%	294	1

HTC One M8	0.1%	322	2
Huawei Honor 7X	0.1%	308	1
Huawei Sensa	0.1%	602	1
LG G Pad	0.1%	490	1
LG Phoenix 3	0.1%	434	1
LG Tribute Dynasty	0.1%	518	1
Lush Mint	0.1%	308	1
Motorola Moto X Style	0.1%	238	1
OnePlus 5	0.1%	658	1
Samsung Galaxy J7 V	0.1%	336	1
ZTE Avid Trio	0.1%	364	1
ZTE Warp 7	0.1%	630	1
Alcatel Revvl	0.0%	196	1
Huawei Ascend XT	0.0%	98	1
Huawei Ascend XT2	0.0%	98	1
Huawei Honor 8	0.0%	224	1
Kyocera Hydro Reach	0.0%	56	1
