# Supplementary material for: Toward a Mobile Platform for Real-world Digital Measurement of Depression: User-Centered Design, Data Quality, and Behavioral and Clinical Modeling
Source: JMIR Ment Health. 2021 Aug 10;8(8):e27589. doi: 10.2196/27589 (PMC8386379; doi:10.2196/27589)
Supplement: Multimedia Appendix 6 [file mental_v8i8e27589_app6.pdf]

# PHQ-9 Figures

Figure S1 shows the histogram of the 3779 PHQ-9 sum scores received from the participants having minimally sufficient data (n=384) over the course of the 12 week study. It is apparent that the PHQ-9 scores covered the whole range of possible scores (0-27) and that the distribution was resembling a normal distribution, with somewhat higher than expected values at both the extremes.

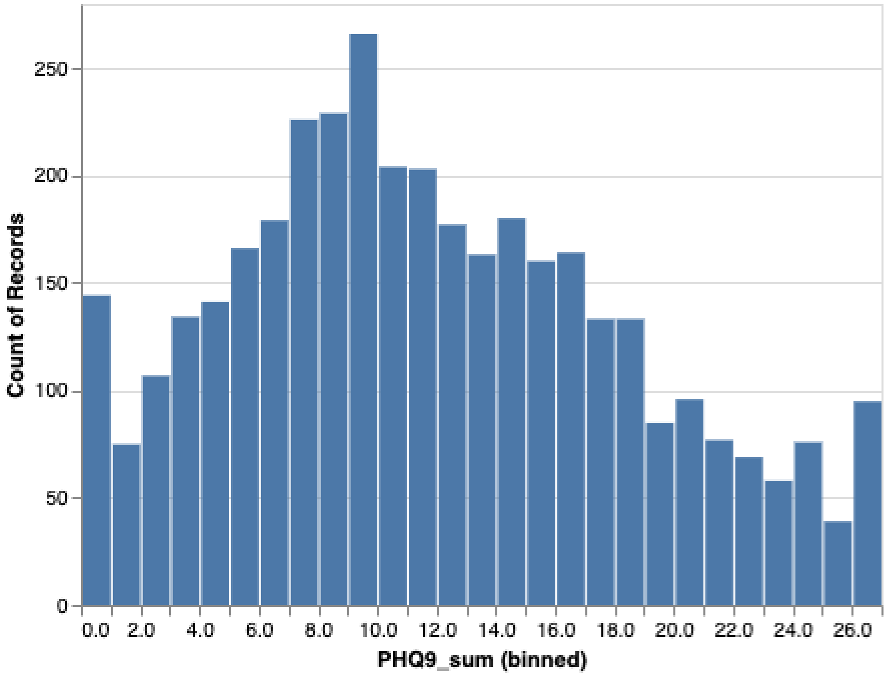


**Figure S1**: Histogram of the 3779 PHQ-9 sum scores received from 384 unique participants over the course of the 12 week study.

Figure S2 shows the association between PHQ-9 score and the 11 behavioral features that showed a significant ($P \leq 0.001$, adjusted by Benjamini-Hochberg procedure) Spearman's rho correlation with the PHQ-9 score.

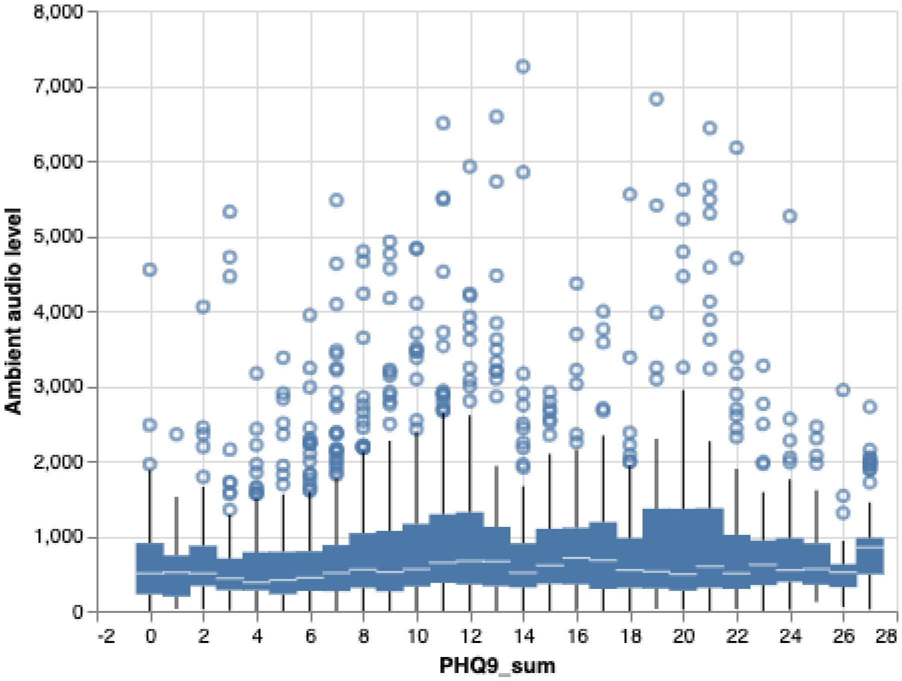

Ambient audio level
PHQ9_sum


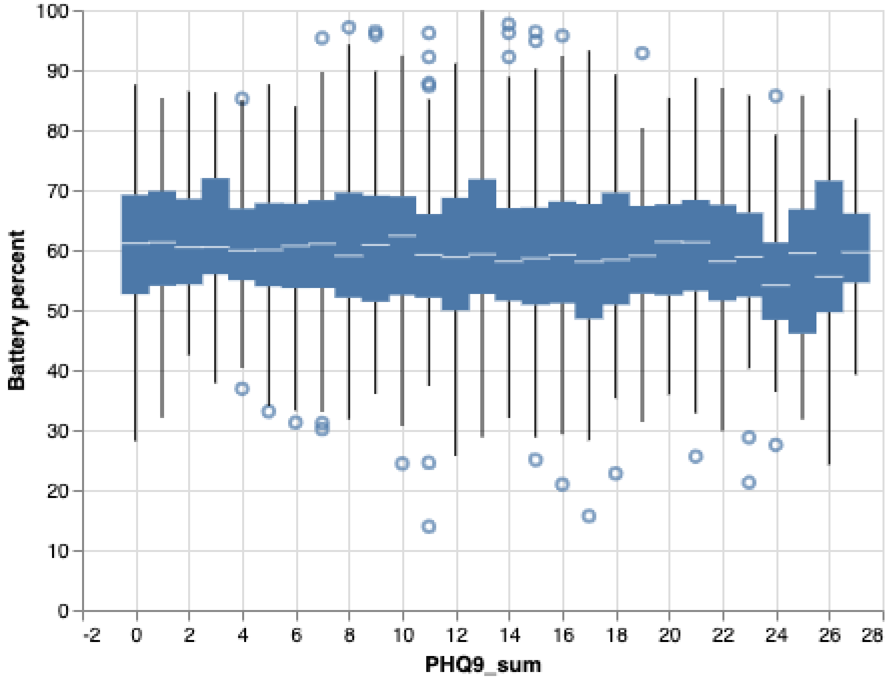

Battery percent
PHQ9_sum

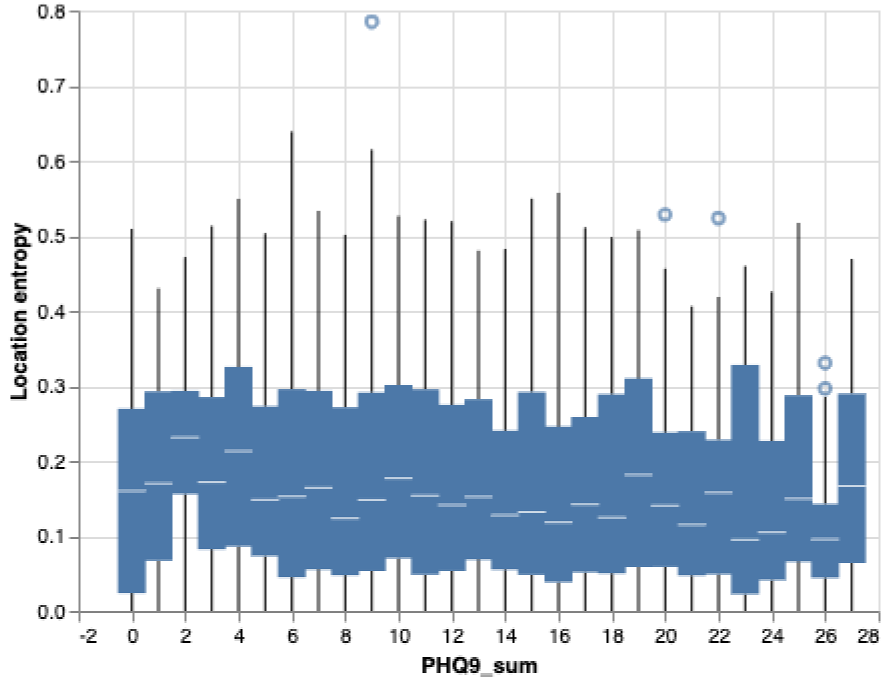

Location entropy
PHQ9_sum
0.8
0.7
0.6
0.5
0.4
0.3
0.2
0.1
0.0
-2
0
2
4
6
8
10
12
14
16
18
20
22
24
26
28


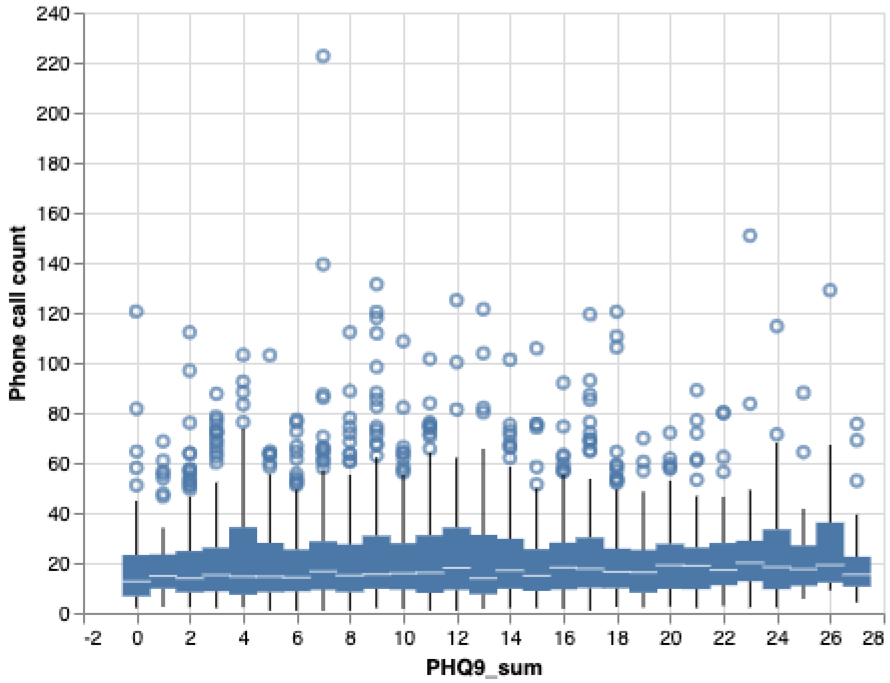

Phone call count
PHQ9_sum
240
220
200
180
160
140
120
100
80
60
40
20
0
-2
0
2
4
6
8
10
12
14
16
18
20
22
24
26
28

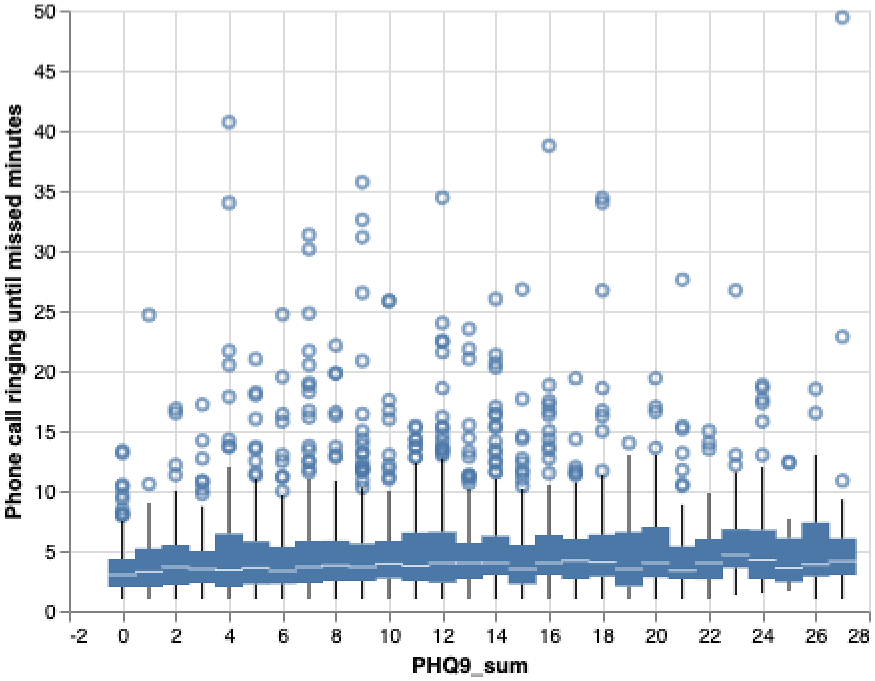
Phone call ringing until missed minutes
PHQ9_sum
-2 0 2 4 6 8 10 12 14 16 18 20 22 24 26 28
0 5 10 15 20 25 30 35 40 45 50

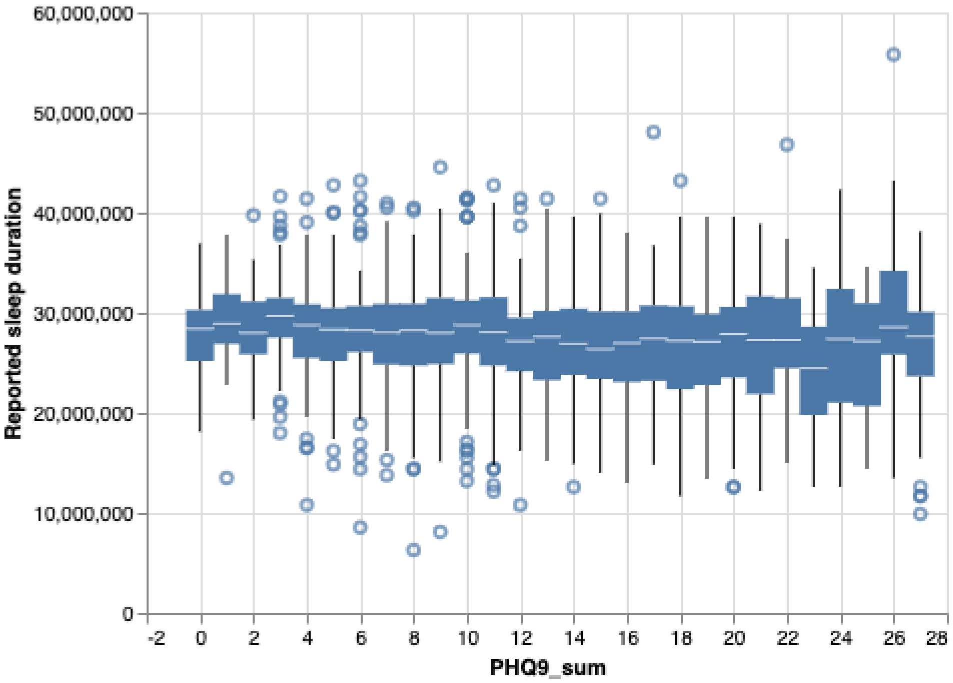
Reported sleep duration
PHQ9_sum
-2 0 2 4 6 8 10 12 14 16 18 20 22 24 26 28
0 10,000,000 20,000,000 30,000,000 40,000,000 50,000,000 60,000,000

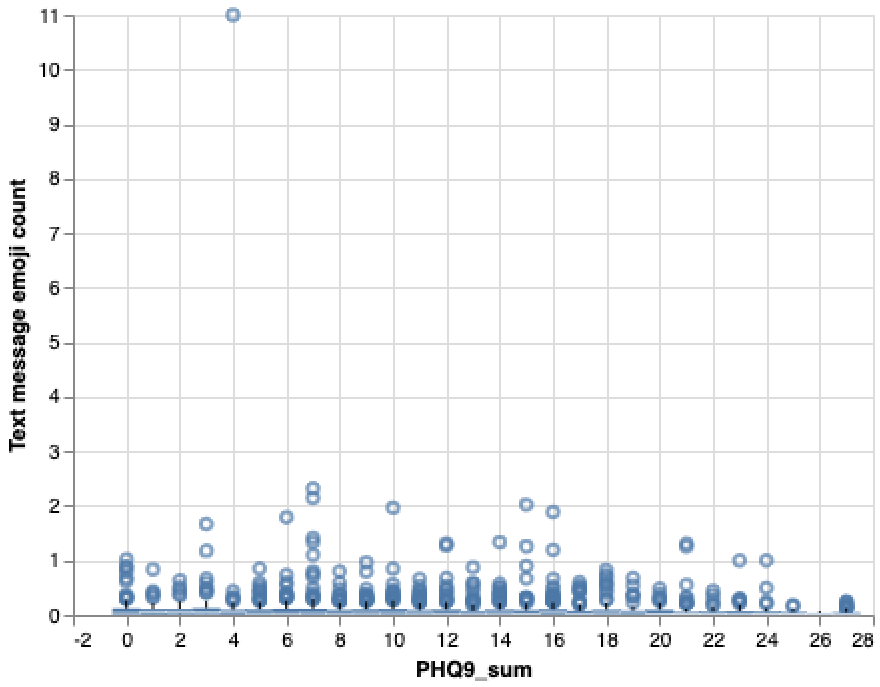

Text message emoji count
PHQ9_sum


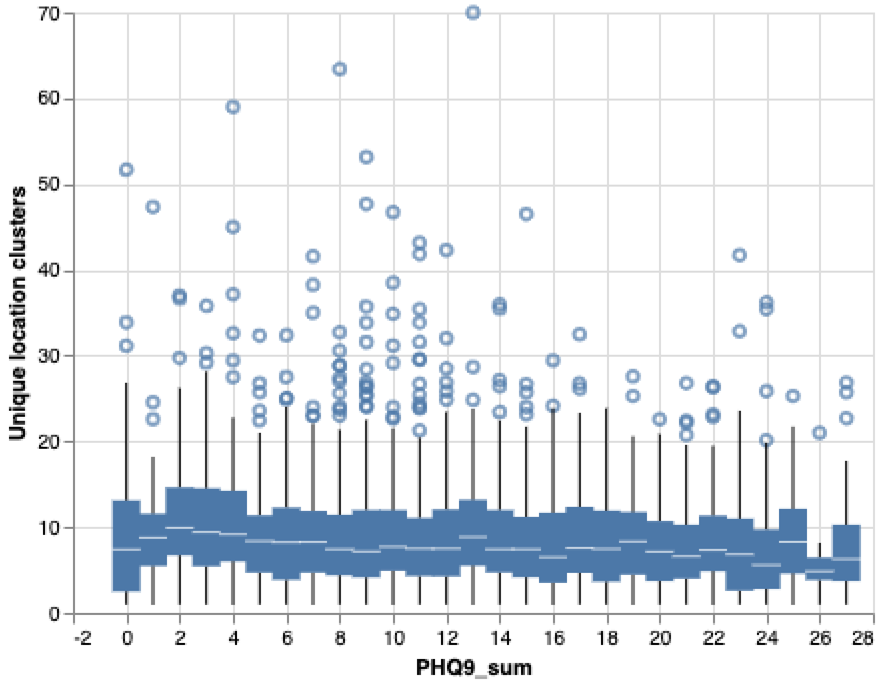

Unique location clusters
PHQ9_sum

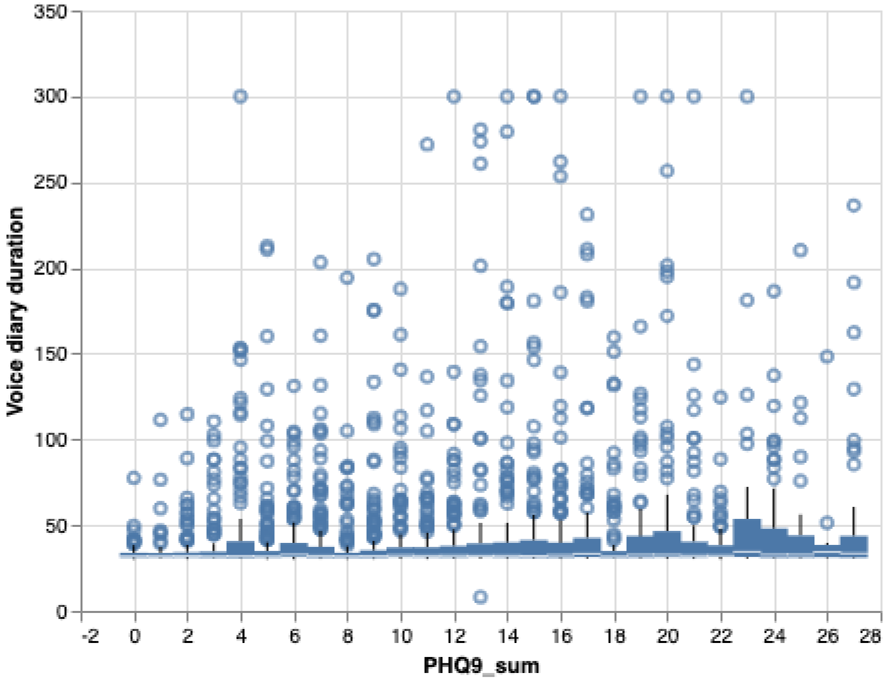

Voice diary duration
PHQ9_sum


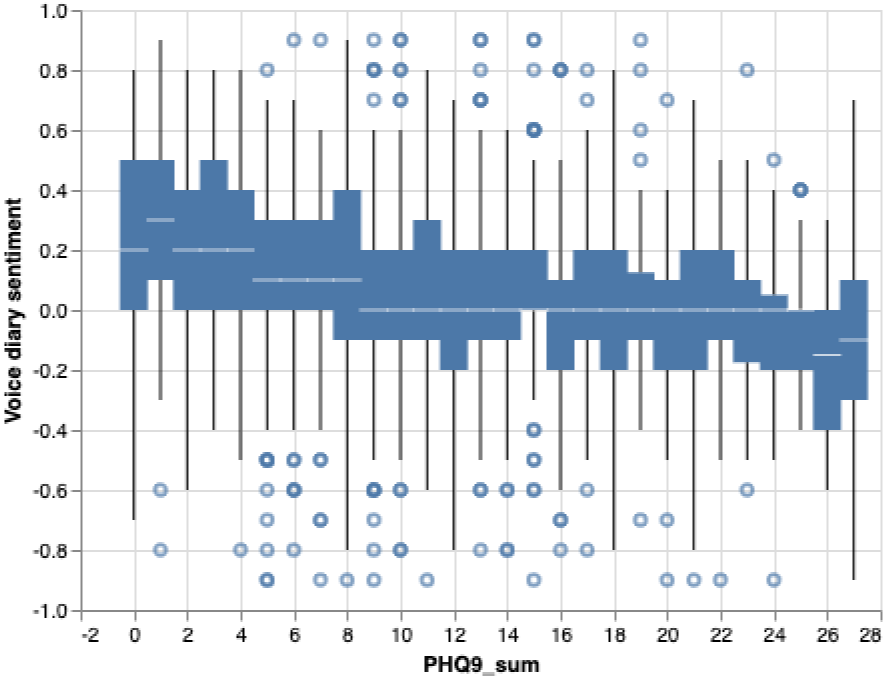

Voice diary sentiment
PHQ9_sum

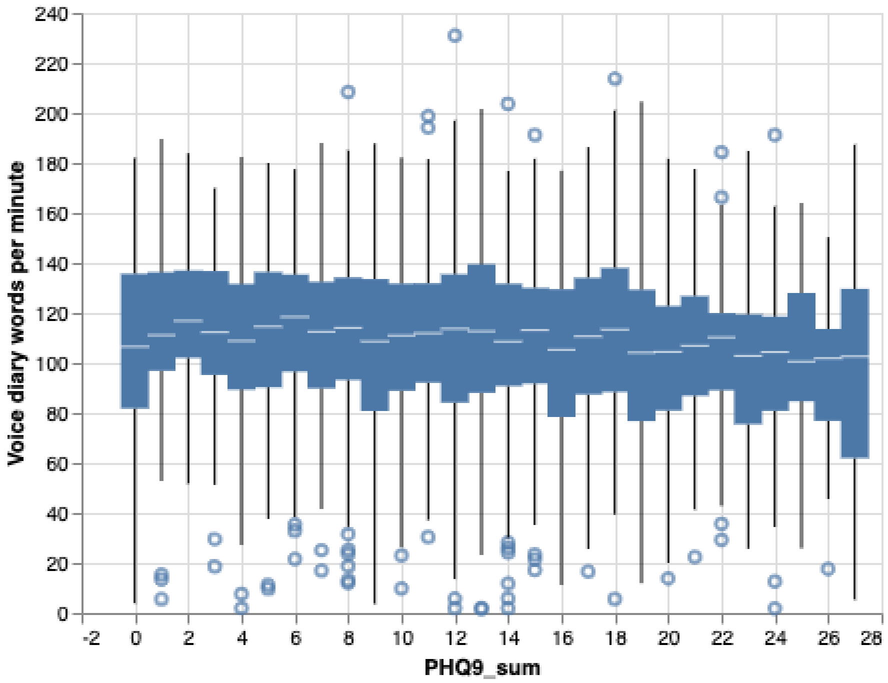


**Figure S2**: For each of the 11 behavioral features that were significantly ($P \leq 0.001$ adjusted by Benjamini-Hochberg procedure) correlated with PHQ-9 score, we show the PHQ-9 score ranging from 0 to 27 on the x-axis, and the values of the respective behavioral features in a box-and-whiskers format on the y-axis. The white line denotes the median. Circles denote outliers defined as any point more than 1.5 IQRs from the box.
