# Supplementary material for: Toward a Mobile Platform for Real-world Digital Measurement of Depression: User-Centered Design, Data Quality, and Behavioral and Clinical Modeling
Source: JMIR Ment Health. 2021 Aug 10;8(8):e27589. doi: 10.2196/27589 (PMC8386379; doi:10.2196/27589)
Supplement: Multimedia Appendix 7 [file mental_v8i8e27589_app7.pdf]

Feature correlation matrix

Table S1 shows the Pearson product-moment correlation matrix for the 34 behavioral features included in the multivariate logistic regression in the main text. The highest correlation is .78 (between audio system volume and audio notification volume), but the majority of correlations is in the small (up to .30) range.

Table S1: Pearson product-moment correlation matrix for the 34 behavioral features.

	Ambient audio level	Ambient light level	App usage missing	Audio notification volume	Audio system volume	Battery percent	Charging minutes	Communication apps usage	Incoming phone call duration	Location entropy	Location variance	Nearby WiFi networks count	Number of WiFi networks	Outgoing phone call duration	Outgoing text message sentiment score	Phone call count	Phone call ringing until missed minutes	Phone screen on minutes	Physically active minutes	Reported sleep duration	Reported sleep duration missing	Ring volume	Social apps usage	Text message body size	Text message count	Text message emoji count	Time spent at home	Time spent at hospital	Unique location clusters	Voice diary duration	Voice diary pauses duration	Voice diary sentiment	Voice diary words per minute	Wellness apps usage
Ambient audio level	1.00	-.03	.00	.09	.06	-.14	-.04	-.01	.00	.03	-.02	-.10	.06	-.01	.00	.13	.06	.12	.08	.00	-.01	.09	-.03	-.06	.06	-.01	-.07	-.05	.05	-.07	-.02	-.01	-.01	-.06
Ambient light level	-.03	1.00	.00	-.04	-.05	-.04	-.04	.06	.01	.23	.02	-.10	-.07	.04	.03	.09	.03	.00	.12	.00	-.02	-.07	-.01	-.08	.05	.01	-.17	-.01	.27	-.06	.09	.01	.00	.04
App usage missing	.00	.00	1.00	-.04	-.01	-.01	-.05	.00	-.04	.03	-.01	-.03	.00	-.03	-.04	-.05	-.06	-.05	-.01	.05	.02	-.05	-.02	-.01	-.01	-.01	-.02	-.05	.01	.05	-.01	-.01	-.02	-.02
Audio notification volume	.09	-.04	-.04	1.00	.78	.03	.06	.00	.09	-.10	-.03	.02	.01	.09	-.01	.17	.10	.05	-.06	-.06	-.02	.74	-.04	.02	.02	.02	.15	-.03	-.02	.03	-.07	.02	-.04	.03
Audio system volume	.06	-.05	-.01	.78	1.00	.00	.02	-.01	.07	-.16	-.03	.01	.00	.08	-.01	.14	.07	.02	-.09	-.04	-.03	.72	-.06	.07	.01	-.01	.14	-.01	-.07	.01	-.07	.03	-.02	.01
Battery percent	-.14	-.04	-.01	.03	.00	1.00	.37	-.02	-.04	.02	-.01	.08	.01	-.05	.02	-.17	-.07	-.07	-.03	.01	.02	.00	-.01	.02	-.06	.01	.05	.07	.03	.07	-.01	-.03	-.01	.08
Charging minutes	-.04	-.04	-.05	.06	.02	.37	1.00	.02	.03	.06	.00	.08	-.08	-.01	-.02	-.11	.01	.19	-.03	-.01	-.08	.05	.06	-.02	-.01	-.02	-.03	-.01	.07	.07	.01	-.04	-.01	.05
Communication apps usage	-.01	.06	.00	.00	-.01	-.02	.02	1.00	.01	.16	.00	.06	-.01	.03	.01	.01	-.01	.10	.05	-.03	-.07	-.05	.61	-.05	.10	-.03	-.03	.02	.14	.01	-.02	-.01	.03	.54
Incoming phone call duration	.00	.01	-.04	.09	.07	-.04	.03	.01	1.00	-.05	-.02	.01	.04	.41	.03	.32	.10	.10	.00	-.03	-.06	.11	-.03	.00	.10	.16	.03	.01	-.01	.03	-.03	.01	.00	-.02
Location entropy	.03	.23	.03	-.10	-.16	.02	.06	.16	-.05	1.00	.07	-.04	-.07	.02	.06	-.03	-.06	-.13	.27	-.02	.07	-.11	.20	-.17	.13	-.03	-.44	.11	.66	.09	.06	-.01	-.03	.12
Location variance	-.02	.02	-.01	-.03	-.03	-.01	.00	.00	-.02	.07	1.00	.00	.00	-.02	.00	-.02	-.01	-.03	.05	.01	.06	-.03	-.02	.01	-.01	-.01	-.08	.02	.14	-.01	-.01	.03	.03	-.03
Nearby WiFi networks count	-.10	-.10	-.03	.02	.01	.08	.08	.06	.01	-.04	.00	1.00	.41	-.05	.01	-.08	.00	-.08	-.07	-.01	-.01	.03	.06	.03	-.14	.00	.37	-.09	-.09	.03	-.07	.02	-.01	.04
Number of WiFi networks	.06	-.07	.00	.01	.00	.01	-.08	-.01	.04	-.07	.00	.41	1.00	.00	.05	-.04	-.07	-.08	-.02	.07	.02	.00	-.02	.00	-.07	.02	.18	-.05	-.06	.04	-.04	.03	.02	-.05
Outgoing phone call duration	-.01	.04	-.03	.09	.08	-.05	-.01	.03	.41	.02	-.02	-.05	.00	1.00	-.01	.55	.12	.08	.12	-.01	-.03	.12	-.05	.01	.19	.03	.00	.05	.05	.03	-.01	.01	-.01	-.03
Outgoing text message sentiment score	.00	.03	-.04	-.01	-.01	.02	-.02	.01	.03	.06	.00	.01	.05	-.01	1.00	-.09	-.04	-.06	-.01	-.03	.02	-.01	.03	-.05	-.01	.06	-.04	.02	.04	.01	-.03	.09	.03	.02
Phone call count	.13	.09	-.05	.17	.14	-.17	-.11	.01	.32	-.03	-.02	-.08	-.04	.55	-.09	1.00	.46	.13	.11	-.05	.01	.19	-.13	-.06	.17	-.02	-.01	-.03	.10	-.02	.00	-.01	-.06	-.11
Phone call ringing until missed minutes	.06	.03	-.06	.10	.07	-.07	.01	-.01	.10	-.06	-.01	.00	-.07	.12	-.04	.46	1.00	.03	.03	-.02	-.02	.05	-.10	.00	.08	-.04	.06	-.04	.03	-.05	.03	-.04	-.03	-.10
Phone screen on minutes	.12	.00	-.05	.05	.02	-.07	.19	.10	.10	-.13	-.03	-.08	-.08	.08	-.06	.13	.03	1.00	.00	-.09	-.04	.05	.06	-.04	.18	.02	.02	.04	.00	.00	-.01	-.01	-.02	.00

	Ambient audio level	Ambient light level	App usage missing	Audio notification volume	Audio system volume	Battery percent	Charging minutes	Communication apps usage	Incoming phone call duration	Location entropy	Location variance	Nearby WiFi networks count	Number of WiFi networks	Outgoing phone call duration	Outgoing text message sentiment score	Phone call count	Phone call ringing until missed minutes	Phone screen on minutes	Physically active minutes	Reported sleep duration	Reported sleep duration missing	Ring volume	Social apps usage	Text message body size	Text message count	Text message emoji count	Time spent at home	Time spent at hospital	Unique location clusters	Voice diary duration	Voice diary pauses duration	Voice diary sentiment	Voice diary words per minute	Wellness apps usage
Physically active minutes	.08	.12	-.01	-.06	-.09	-.03	-.03	.05	.00	.27	.05	-.07	-.02	.12	-.01	.11	.03	.00	1.00	-.05	.06	-.08	.05	-.06	.11	-.03	-.17	.12	.22	-.02	.06	-.02	.03	.02
Reported sleep duration	.00	.00	.05	-.06	-.04	.01	-.01	-.03	-.03	-.02	.01	-.01	.07	-.01	-.03	-.05	-.02	-.09	-.05	1.00	-.04	-.07	.02	.02	.09	-.01	.01	.06	-.03	-.06	.01	.06	.04	.00
Reported sleep duration missing	-.01	-.02	.02	-.02	-.03	.02	-.08	-.07	-.06	.07	.06	-.01	.02	-.03	.02	.01	-.02	-.04	.06	-.04	1.00	-.03	-.04	-.01	.03	.03	-.10	.03	.05	.03	.04	-.01	-.03	-.11
Ring volume	.09	-.07	-.05	.74	.72	.00	.05	-.05	.11	-.11	-.03	.03	.00	.12	-.01	.19	.05	.05	-.08	-.07	-.03	1.00	-.10	.07	.00	.02	.15	-.03	-.03	.05	-.09	-.02	-.05	-.02
Social apps usage	-.03	-.01	-.02	-.04	-.06	-.01	.06	.61	-.03	.20	-.02	.06	-.02	-.05	.03	-.13	-.10	.06	.05	.02	-.04	-.10	1.00	-.07	.14	-.03	-.07	.02	.07	.02	-.03	.02	.07	.50
Text message body size	-.06	-.08	-.01	.02	.07	.02	-.02	-.05	.00	-.17	.01	.03	.00	.01	-.05	-.06	.00	-.04	-.06	.02	-.01	.07	-.07	1.00	-.29	-.03	.16	.04	-.14	.08	-.08	-.02	.02	-.03
Text message count	.06	.05	-.01	.02	.01	-.06	-.01	.10	.10	.13	-.01	-.14	-.07	.19	-.01	.17	.08	.18	.11	.09	.03	.00	.14	-.29	1.00	.00	-.17	.01	.13	-.08	.03	.08	.03	.05
Text message emoji count	-.01	.01	-.01	.02	-.01	.01	-.02	-.03	.16	-.03	-.01	.00	.02	.03	.06	-.02	-.04	.02	-.03	-.01	.03	.02	-.03	-.03	.00	1.00	.01	-.01	-.02	.03	-.02	.04	.00	-.03
Time spent at home	-.07	-.17	-.02	.15	.14	.05	-.03	-.03	.03	-.44	-.08	.37	.18	.00	-.04	-.01	.06	.02	-.17	.01	-.10	.15	-.07	.16	-.17	.01	1.00	-.05	-.26	-.03	-.04	-.01	.00	.01
Time spent at hospital	-.05	-.01	-.05	-.03	-.01	.07	-.01	.02	.01	.11	.02	-.09	-.05	.05	.02	-.03	-.04	.04	.12	.06	.03	-.03	.02	.04	.01	-.01	-.05	1.00	.07	-.01	.01	-.02	.01	.04
Unique location clusters	.05	.27	.01	-.02	-.07	.03	.07	.14	-.01	.66	.14	-.09	-.06	.05	.04	.10	.03	.00	.22	-.03	.05	-.03	.07	-.14	.13	-.02	-.26	.07	1.00	.04	.03	.03	-.03	.07
Voice diary duration	-.07	-.06	.05	.03	.01	.07	.07	.01	.03	.09	-.01	.03	.04	.03	.01	-.02	-.05	.00	-.02	-.06	.03	.05	.02	.08	-.08	.03	-.03	-.01	.04	1.00	-.02	-.09	-.38	-.01
Voice diary pauses duration	-.02	.09	-.01	-.07	-.07	-.01	.01	-.02	-.03	.06	-.01	-.07	-.04	-.01	-.03	.00	.03	-.01	.06	.01	.04	-.09	-.03	-.08	.03	-.02	-.04	.01	.03	-.02	1.00	-.03	-.27	-.05
Voice diary sentiment	-.01	.01	-.01	.02	.03	-.03	-.04	-.01	.01	-.01	.03	.02	.03	.01	.09	-.01	-.04	-.01	-.02	.06	-.01	-.02	.02	-.02	.08	.04	-.01	-.02	.03	-.09	-.03	1.00	.09	-.03
Voice diary words per minute	-.01	.00	-.02	-.04	-.02	-.01	-.01	.03	.00	-.03	.03	-.01	.02	-.01	.03	-.06	-.03	-.02	.03	.04	-.03	-.05	.07	.02	.03	.00	.00	.01	-.03	-.38	-.27	.09	1.00	.06
Wellness apps usage	-.06	.04	-.02	.03	.01	.08	.05	.54	-.02	.12	-.03	.04	-.05	-.03	.02	-.11	-.10	.00	.02	.00	-.11	-.02	.50	-.03	.05	-.03	.01	.04	.07	-.01	-.05	-.03	.06	1.00
